# Sense of self impacts spatial navigation and hexadirectional coding in human entorhinal cortex

**DOI:** 10.1038/s42003-022-03361-5

**Published:** 2022-05-02

**Authors:** Hyuk-June Moon, Baptiste Gauthier, Hyeong-Dong Park, Nathan Faivre, Olaf Blanke

**Affiliations:** 1 Center of Neuroprosthetics, Faculty of Life Sciences, Swiss Federal Institute of Technology (École Polytechnique Fédérale de Lausanne, EPFL), Geneva, Switzerland; 2 Brain Mind Institute, Faculty of Life Sciences, Swiss Federal Institute of Technology (École Polytechnique Fédérale de Lausanne, EPFL), Lausanne, Switzerland; 3 Center for Bionics, Biomedical Research Division, Korea Institute of Science and Technology (KIST), Seoul, South Korea; 4 Graduate Institute of Mind, Brain and Consciousness, Taipei Medical University, Taipei, Taiwan; 5 Brain and Consciousness Research Centre, Shuang-Ho Hospital, New Taipei City, Taiwan; 6 University Grenoble Alpes, University Savoie Mont Blanc, CNRS, LPNC, Grenoble, France; 7 Department of Neurology, University Hospital Geneva, Geneva, Switzerland

**Keywords:** Consciousness, Spatial memory, Navigation

## Abstract

Grid cells in entorhinal cortex (EC) encode an individual’s location in space and rely on environmental cues and self-motion cues derived from the individual’s body. Body-derived signals are also primary signals for the sense of self and based on integrated sensorimotor signals (proprioceptive, tactile, visual, motor) that have been shown to enhance self-centered processing. However, it is currently unknown whether such sensorimotor signals that modulate self-centered processing impact grid cells and spatial navigation. Integrating the online manipulation of bodily signals, to modulate self-centered processing, with a spatial navigation task and an fMRI measure to detect grid cell-like representation (GCLR) in humans, we report improved performance in spatial navigation and decreased GCLR in EC. This decrease in entorhinal GCLR was associated with an increase in retrosplenial cortex activity, which was correlated with participants’ navigation performance. These data link self-centered processes during spatial navigation to entorhinal and retrosplenial activity and highlight the role of different bodily factors at play when navigating in VR.

## Abstract (web summary)

fMRI recordings from human participants manipulating a virtual avatar in a spatial navigation task show higher performance, but lower grid cell-like activity, compared to the navigation where no avatar is visible.

## Introduction

The discovery of grid cells in rodent entorhinal cortex (EC) has shed new light on the neural mechanisms of spatial representation^1,2^. Grid cells are place-modulated neurons believed to represent the location of an individual and are defined by characteristic spatial firing field maps corresponding to hexagonal grid patterns that tile a given environment^3,4^. Entorhinal grid cell activity is modulated by sensory cues from the environment as well as by motion-related cues from the individual (i.e., self-motion cues)^1,2,5,6^. While the field maps of grid cells have been shown to depend on distal landmarks and field boundaries^1,7^, their periodicity is maintained in darkness and across different environments and landmark changes^1,8^. This suggests that movement-related signals from the body also provide a considerable input to grid cells. Indeed, subsequent studies demonstrated the primary importance of such self-motion cues from the body for generating and maintaining grid representations^9–11^. Overall, these findings support the proposal that grid cells keep track of an individual’s location in space by relying on both self-motion cues and environmental sensory information^5,12^.

Self-motion cues are body-derived cues based on sensory and motor signals from the individual’s body during spatial navigation and include proprioceptive, tactile, vestibular, and motor signals^2,5,13^. Under normal conditions, the self is bound to the location of the physical body and experienced at the place occupied by the body. Although this association between self and body is a central feature of self-consciousness, captured by the concept of bodily self-consciousness (BSC)^14–16^, it can be modulated experimentally. Thus, recent research using virtual reality (VR) has shown that non-ordinary BSC states, such as illusory self-identification for an avatar or virtual body, can be induced by employing the online manipulation of sensory and motor signals from the individual’s body^15,17^. In some BSC paradigms^18–20^, participants receive tactile stimulation while viewing an avatar as seen from behind and projected in front of them that receives the same tactile stimulation (third-person viewpoint). In other BSC paradigms, the avatar is shown from a first-person viewpoint, while participants are exposed to visuotactile stimulation on the chest^21,22^. Both types of BSC studies showed that such enhanced self-centered processing results from integrated body-derived stimuli (proprioception, vision, touch) that are spatially and temporally congruent. Recent BSC studies further demonstrated that such enhanced self-centered processing associated with BSC changes also impact egocentric spatial processes (size perception^23–26^, spatial semantic distance^27^), linking BSC not just to self-centered processing, but also to spatial processing. However, whereas the key importance of body-derived sensorimotor signals in grid cells is well documented, it is currently unknown whether and how online sensorimotor bodily stimulations in VR, which have been shown to enhance self-centered processing linked to BSC, impact entorhinal grid cell activity. Here, we sought to investigate whether sensorimotor signals that enhance self-centered processing in a BSC paradigm implemented during spatial navigation in VR would modulate grid cell-like activity in EC.

While human grid cells have only rarely been described using single-unit recordings in epilepsy patients^28,29^, a method based on functional magnetic resonance imaging (fMRI) detecting a specific pattern in parametric BOLD (blood-oxygen-level-dependent) signal changes, the so-called grid cell-like representation (GCLR), has been proposed to reflect the activity of human grid cell populations^30–35^. GCLR is assumed to capture BOLD activity from populations of conjunctive grid cells in human EC^36^, characterized by heading-direction-dependent BOLD signal modulation with sixfold rotational symmetry following hexagonal grids of grid cells. Thus, the magnitude of hexadirectional BOLD signal modulation, GCLR, has been suggested as a proxy grid cell activity in humans^5,30,31^. To investigate whether enhanced self-centered processing impacts grid cell-like activity in EC, we designed a sensorimotor VR task and manipulated BSC while our participants performed a classical spatial navigation task as used in previous fMRI research^30,31^ that allowed us to assess spatial navigation performance and calculate GCLR. We experimentally enhanced self-centered processing with a BSC paradigm by showing an online avatar from the first-person viewpoint that was spatially congruent with the participants’ body. We hypothesized that this condition would strengthen self-centered bodily processing and enhance entorhinal GCLR based on the reported importance of self-centered input to grid cells^1,10,11^. We further expected improved spatial navigation performance in the BSC-enhanced condition. Performance and GCLR were compared to an experimental condition without enhancement of self-centered bodily processing (i.e., no avatar shown, classical spatial navigation paradigm used in fMRI). We, thus, assessed the impact of BSC on grid cell-like activity in a condition of enhanced self-centered processing by manipulating our participants’ self-identification with the avatar shown during navigation.

## Results

### Avatar-related changes in BSC enhance spatial navigation performance

**Fig. 1:**
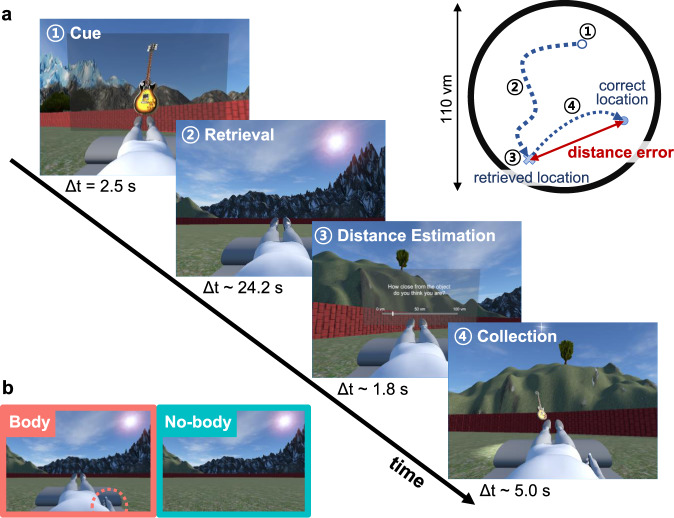
Spatial navigation task and experimental BSC conditions. **a** The spatial navigation task consisted of six sessions with two experimental conditions. Each session started with an encoding phase, in which participants had to memorize the locations of three objects. Following encoding, participants performed 14 trials with the following steps: (1) Cue: a target object was provided (2.5 s); (2) Retrieval: they had to recall and reach the original object location (self-paced, average 24.2 s); (3) Distance estimation: they estimated the distance error they committed (self-paced, average 1.8 s); (4) Collection: a target object appeared at its original location and participants were asked to navigate to it (self-paced, average 5.0 s). **b** In the Body condition, a body-shaped avatar (congruent with the posture and hand motion of the participant in the scanner) was seen by participants as part of the virtual scene during the entire procedure. In the No-body condition, the same scenes were displayed, but without the avatar (as is usually done during spatial navigation studies). Δt mean duration, vm virtual meter.

We adopted a spatial navigation task from previous fMRI studies^30,31^ to assess spatial navigation performance and BSC, and to calculate GCLR (Fig. 1a; see Methods). Each session started with an encoding phase, in which participants had to memorize the locations of three objects. Following encoding, for each trial, a cue indicated a specific target object that participants had to recall and reach by navigating to it in the arena. At the end of each retrieval trial, the distance between the recalled location and its correct location (i.e., “distance error”) was determined. Navigation trace length and navigation time were also recorded in order to quantify spatial navigation performance. To assess the influence of BSC (self-identification) on grid cell-like activity as reflected in GCLR, we designed two experimental conditions and induced different levels of self-identification with the avatar by providing different online sensorimotor stimulation during the task. In the Body condition, supine participants saw, from their first-person viewpoint, a supine virtual avatar, which was spatially congruent with their own body position. As shown in Fig. 1a, we also showed the virtual right hand of the avatar (and a virtual joystick) that carried out the same movements as the participant’s right hand on the physical joystick in the scanner. Such spatial congruency between the participant’s body and the avatar’s body (or alignment of bodily reference frames; see methods for further detail) has been investigated in previous BSC research and shown to induce higher levels of self-identification with the avatar^37,38^. By contrast, the No-body condition did neither contain an avatar nor the right-hand movements and served as a control condition, for which we expected no or less self-identification as compared to the Body condition. Importantly, the No-body condition is identical to most previous human GCLR studies^30,31^.

**Fig. 2:**
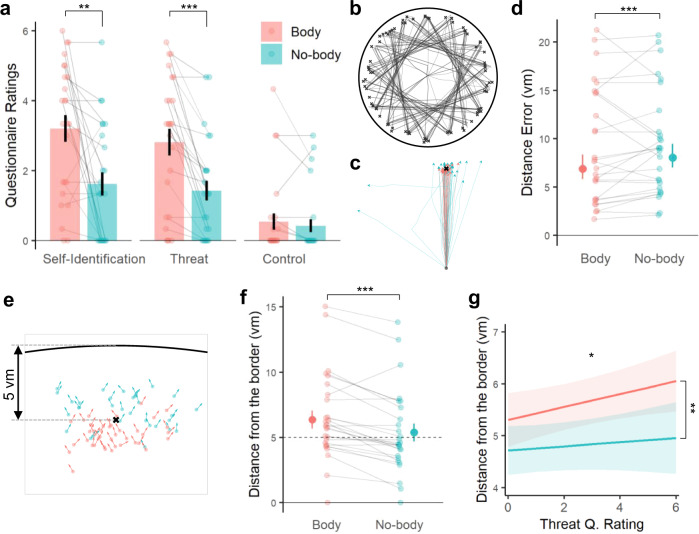
Enhanced self-identification was improved spatial navigation performance in the Body condition. **a** Ratings of the questionnaire confirmed the effect of the experimental modulation on BSC (*n* = 25). Self-identification (Q1: *p* = 2.53e-03) and experienced threat (Q2: *p* = 1.42e-04) were rated significantly higher in the Body vs. the No-body condition. Each bar represents the condition-wise mean across participants, while each error bar indicates a standard error. **b** Exemplary traces from a participant during the spatial navigation task. **c** Overlay of the navigation traces per condition during the retrieval phase of the same participant (traces were rotated and shifted according to the starting and the target location, in order to better visualize the difference in distance errors and navigation efficiency). **d** Participants showed better spatial memory precision, indexed by lower distance errors from the correct retrieval targets (*n* = 27; *p* = 8.25e-04). In the graph, a point with whiskers indicates distance error and its 95% confidence interval estimated by the mixed-effect model. Each smaller dot represents the median of an individual participant per condition. **e** The arrows display the trial-by-trial reached locations and heading directions of an exemplary participant. Locations are plotted relative to the correct target point (“x”), and the arena’s border (black bold line). **f** Participants stopped farther away from the arena’s border compared to the No-body condition during which no virtual avatar was presented (*n* = 27; *p* = 4.80e-13). A point with whiskers indicates distance error and its 95% confidence interval estimated by the mixed-effect model. Each smaller dot represents the median of an individual participant per condition. **g** Mixed-effect model slopes relating Threat (Q2) to the distance from the border in the two conditions (*n* = 25; *p* = 0.026 without correction), while taking into account the condition-wise difference (*p* = 9.67e-3). For the pannels **d**, **f** participant-wise median values were visualized, while the statistical analysis was performed with the trial-wise values through a dedicated mixed model. *0.01 < = *p* < 0.05, **0.001 < = *p* < 0.01, ***p < 0.001.

We assessed BSC by asking participants to rate their self-identification with the avatar (Q1: Self-Identification), to rate experienced threat (in response to a virtual knife that was seen as approaching the part of the arena where the virtual avatar was located) (Q2: Threat; see Methods and Supplementary Fig. 1c), and also assessed two control items (Q3, Q4; see Methods). As predicted, ratings to Q1 and Q2 were higher in the Body vs. No-body condition (paired two-sided Wilcoxon signed-rank test, Q1: *Z* = 3.02, *r* = 0.60, *p* = 2.53e-03; Q2: *Z* = 3.80, *r* = 0.76, *p* = 1.42e-04, *n* = 25; Fig. 2a), indicating that our manipulation was effective in modulating BSC. Although we cannot exclude the influence of the demand characteristics while our participants were responding to the questionnaire, they did not give different ratings in the control questions, making it less likely that the observed effects were merely driven by the biases (i.e., no difference across conditions in control questions; Q3: *Z* = 0.57, *r* = 0.11, *p* = 0.569 Q4: *Z* = 1.05, *r* = 0.21, *p* = 0.293; see Methods; Supplementary Fig. 1a). Post-experiment debriefing confirmed these results (see Methods; Supplementary Fig. 1b).

To assess the influence of the BSC modulation on spatial navigation and memory, we compared spatial navigation/memory performance during both conditions. All participants were able to navigate in the virtual environment and complete the task while being scanned in the MRI scanner (Fig. 2b). Interestingly, participants showed better spatial memory precision as indexed by lower distance errors in the Body vs. No-body condition (mixed-effects regression; df = 1, *F* = 11.18, *p* = 8.25e-04, *n* = 27; Fig. 2d). Navigation efficiency was also improved, as participants carried out shorter paths in the Body condition vs. the No-body condition (df = 1, *F* = 8.46, *p* = 3.81e-03; Supplementary Fig. 2a), while average navigation time did not differ across conditions (df = 1, *F* = 0.21, *p* = 0.648; Supplementary Fig. 2b). These results demonstrate that the Body condition induces higher self-identification with the virtual avatar (as indexed by Q1 and Q2), as well as higher spatial navigation/memory performance in the virtual environment.

Of note, participants stopped navigating significantly farther from the arena’s border in the Body condition (i.e., the condition where they see a self-identified avatar in front of them) compared to the No-body condition (df = 1, *F* = 52.29, *p* = 4.80e-13, *n* = 27; Fig. 2e, f). This navigational difference was consistently observed in 22 out of 27 participants. Our finding is compatible with spatial changes, referred to as a drift in self-location toward a self-identified avatar, reported by previous research on BSC using different behavioral measures^18,19,39,40^. Thus, when self-identifying with the avatar seen in front of them, our participants stopped before reaching the intended destination, in turn, farther from the border (Fig. 2e). This link between the drift in spatial navigation and BSC was further confirmed by the significant relationship between the drift and BSC ratings (i.e., Threat; Q2): the more participants felt threatened by the virtual knife directed to where the avatar was, the farther they stopped away from the arena’s border before reaching the target (1.00 ± 0.27 vm; predicted by mixed-effects regression; df = 1, *F* = 11.23, *p* = 0.0263 without the multiple testing correction for two questions (Q1&Q2), *n* = 25; Fig. 2g). Of note, we did not find a significant correlation between Q1 and the drift. We reiterate that spatial memory precision was higher in the Body condition, despite the fact that, on average, participants stopped farther from the optimal target point (5 vm) in the Body condition (6.38 ± 0.70 vm away from the border; Fig. 2f-red) compared to the No-body condition (5.38 ± 0.67 vm; Fig. 2f-blue). Thus, although it could be argued that the drift may worsen the distance error in the Body condition (participants stopped too early), the opposite was the case for overall spatial navigation performance. Two types of angular errors were calculated in order to further assess spatial memory, eliminating the influence of the drift in self-location effect on the spatial memory precision. The results show that both types of the angular errors were also significantly lower in the Body condition (type 1: mixed-effects regression; df = 1, *F* = 5.397, *p* = 0.020, *n* = 27; type 2: df = 1, *F* = 7.21; *p* = 7.25e-3; Supplementary Fig. 2c).

To summarize, these behavioral results show that the Body condition was characterized by higher self-identification with the avatar and drift in self-location influencing navigational behavior, and, importantly, by an improvement in spatial navigation performance.

### Grid cell-like representation decreases when spatial navigation is performed with a self-identified avatar

**Fig. 3:**
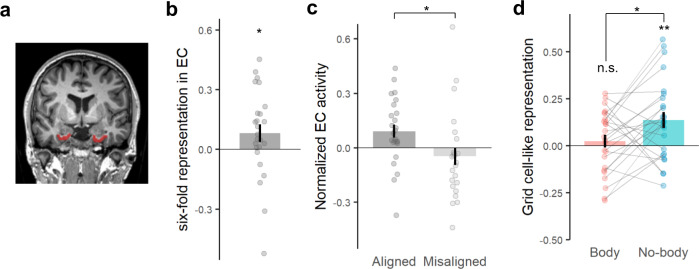
Decrease of entorhinal grid cell-like representation in the Body condition. **a** EC ROI of an exemplary participant **b** Significant sixfold symmetric grid cell-like representation (GCLR) in human entorhinal cortex (EC), calculated for the entire recording session regardless of the experimental condition (*n* = 25; *p* = 0.0128). **c** EC activity during aligned navigation was significantly higher than during misaligned navigation (*n* = 25; *p* = 0.0226). **d** Condition-wise GCLRs were significantly higher in the No-body (standard spatial navigation condition) than the Body condition (*n* = 25; *p* = 0.0236). Notably, condition-wise grid cell-like representations in the Body condition were not significantly greater than zero (Body: *p* = 0.245, No-body: *p* = 2.30e-03), implying that the difference between conditions can be attributed to a reduced GCLR in the Body condition. n.s.: *p* > = 0.05, *0.01 < = *p* < 0.05, **p < 0.01. Each error bar indicates a standard error.

We next assessed whether these changes in BSC and spatial navigation were associated with changes in grid cell activity as reflected by GCLR in EC. As a first step, we confirmed the recruitment of GCLR in our task regardless of the experimental condition, applying previously established methods^30,32,35^. A putative grid orientation ($\varphi$) of each session was estimated with the other five sessions of fMRI images matched with heading direction$\,(\theta )$ information. Based on the calculated grid orientation, the GCLR of each session was determined by the magnitude of the sixfold symmetric fluctuation as a function of the heading direction (see Methods). This analysis revealed a significant hexadirectional BOLD signal modulation, GCLR, in EC when our participants navigated in the virtual environment (sinusoidal regressor: *Z* = 2.23, *r* = 0.45, *p* = 0.0128, Fig. 3b; aligned vs. misaligned contrast: *Z* = 2.00, *r* = 0.40, *p* = 0.0226, *n* = 25, Fig. 3c), replicating earlier data^30^ (also see Supplementary Fig. 3).

Next, we determined GCLR differences between the two conditions by calculating condition-wise GCLR using cross-validation, which was designed to be independent of the other sessions and, in turn, each condition (see Methods). These results confirmed that GCLR was present in the No-body condition (the condition that is similar to conditions used by previous human spatial navigation and GCLR studies; *Z* = 2.83, *r* = 0.57, *p* = 2.30e-03, *n* = 25; Fig. 3d). GCLR was absent in the Body condition (*Z* = 0.69, *r* = 0.14, *p* = 0.245; Fig. 3d), which is the condition with enhanced BSC and spatial navigation performance. Within-subject comparisons between both conditions confirmed significantly lower GCLR in the Body vs. No-body condition (*Z* = 2.26, *r* = 0.45, *p* = 0.0236). We further assessed whether the difference in GCLR between the conditions is related to the difference in the behavioral parameters (e.g., navigated trace length, distance to the border). In-depth control analyses with the resampled datasets that have the inverted or non-significant condition-wise differences in those behavioral parameters again corroborated our results, showing that GCLR was prominent only in the No-body condition regardless of the behavioral parameters (see Supplementary Note 3 and Supplementary Figs. 8, 9).

### RSC activity correlates with improved spatial navigation performance

**Fig. 4:**
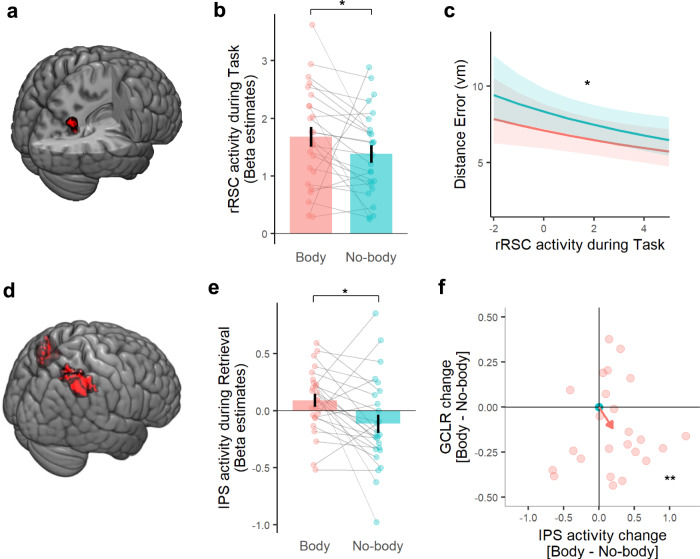
Retrosplenial cortex (RSC) and intraparietal sulcus (IPS) activity were increased in the Body condition. **a** Functional localizer revealed that the right RSC was involved in the spatial navigation task. **b** ROI analysis showed that right RSC was significantly more activated during the task in the Body condition than in the No-body condition (Bonferroni-corrected for five task-related clusters; *n* = 25; *p* = 0.040). **c** The higher right RSC activity during the task phase before they reach the recalled location (i.e., Cue & Retrieval Phase) could predict better spatial memory precision (*n* = 25; *p* = 0.024). **d** Anatomical display of the a priori IPS ROI arguably activated during egocentric processing in link with BSC. **e** IPS activity is significantly greater during navigation in the Body condition (*n* = 25; *p* = 0.015), where sensorimotor bodily signal integration takes place when participants are manipulating the joystick to navigate. This suggests that the experimental modulation of BSC boosted egocentric processes especially relevant to integrating sensorimotor bodily signals. **f** Participant-wise IPS activity changes and GCLR changes in the Body condition with respect to the No-body condition. The plot demonstrates that performing the task with a self-identified avatar reduced GCLR while strengthening the IPS activity (multinomial test: *p* = 5.4e-03, post hoc binomial test: *p* = 3.1e-03, *n* = 24). The red arrow indicates mean changes across participants. *0.01 < *p* < 0.05, **p < 0.01. Each error bar indicates a standard error.

In order to investigate the brain systems possibly accounting for the improved spatial navigation performance in the Body condition, we first assessed the correlation between GCLR and spatial memory precision. However, this was not found to be significant (df = 1, *F* = 0.022, *p* = 0.717, *n* = 25). Even though the grid cell system in EC is known to play a key role in spatial navigation^5,41^, previous human spatial navigation studies showed that other brain regions, such as the retrosplenial cortex (RSC) and parahippocampal gyrus (PHC), are also prominently involved and often closely associated with spatial navigation performance^42–48^. To investigate this in other potentially involved brain regions, we applied whole-brain fMRI analysis (generalized linear model, GLM) and detected five clusters showing significant task-related activations (independently of the experimental conditions), which included the bilateral RSC, bilateral PHC, and right lingual gyrus (LiG) (Fig. 4a, Supplementary Fig. 5a, and Supplementary Table 2), consistently with the existing spatial navigation literature. Comparing activity in each of these five regions of interest (ROIs) between the Body vs. No-body condition during the task phases determining spatial memory precision (i.e., Cue and Retrieval, Fig. 1a), we observed significantly greater activity in right RSC (Fig. 4b; *Z* = 2.65, *r* = 0.53, *p* = 0.040, *n* = 25; Bonferroni-corrected). No significant differences were found in any of the other four regions (bilateral PHC, left RSC, right LiG; Supplementary Fig. 5b). We further observed that higher right RSC activation was associated with better spatial memory precision (characterized by a smaller distance error; Fig. 2d) (df = 1, *F* = 12.11, *p* = 0.024, *n* = 25 Fig. 4c), further linking right RSC to improved spatial navigation performance in the Body condition. The results reveal the prominent implication of RSC in the present task and its contribution to improved spatial navigation performance in the Body condition. In addition, we found that the changes of right RSC activity (between conditions) were related to changes in GCLR activity in EC (Supplementary Fig. 5c), linking both structures in the present task and showing that reduced entorhinal GCLR was associated with increased RSC activity.

### Posterior parietal cortex activity is enhanced during spatial navigation in the Body condition

In an additional control analysis, we assessed whether a core region of BSC, the intraparietal sulcus region (IPS) in the posterior parietal cortex^14,15,49–51^, was differently involved in the two spatial navigation conditions. Importantly, IPS is a core region not only for the integration of multisensory bodily signals and BSC but also for egocentric spatial processing in spatial navigation^46,52,53^. We expected that IPS activity (Fig. 4d; see Methods) would be enhanced in the BSC-enhanced Body condition and that this would be especially the case during navigation because only during navigation did participants receive different online sensorimotor signals (i.e. while participants are navigating by manipulating the joystick). In accordance with our expectation, we found significantly greater IPS activation during navigation (i.e., Retrieval phase) in the Body vs. No-body condition (Fig. 4e; *Z* = 2.44, *r* = 0.49, *p* = 0.0147, n = 25), but not during the Cue phase (*Z* = 0.85, *r* = 0.17, *p* = 0.396). Participant-wise changes in IPS activity, as well as GCLR in EC, demonstrated that IPS activity was increased in the Body vs. No-body condition, while GCLR was decreased in the Body (Fig. 4f). These associated changes, induced by the BSC modulation (i.e., self-identification with the avatar), thereby link both structures: IPS and EC. This was found again only during navigation (multinomial test: *p* = 5.4e-03, post hoc binomial test with H0 probability 0.25: *p* = 3.1e-03, *n* = 24), but not during the non-navigation cue phase (multinomial test; *p* = 0.09). These IPS and GCLR results in associate GCLR attenuation, during spatial navigation with a self-identified avatar, with increased IPS activation in the same condition.

## Discussion

Here we show that signals that are of relevance for BSC impact grid cell-like activity by inducing a state of enhanced self-centered processing (enhanced self-identification with an avatar during spatial navigation; i.e., Body condition) during a spatial navigation task. The Body condition was associated with improved spatial navigation performance and decreased GCLR in EC, which has been proposed to reflect the activity of grid cell populations^30–35^. This unexpected decrease in entorhinal GCLR was associated with an increase in RSC activity, another major spatial navigation region^45,54,55^. Moreover, RSC activity, stronger during the Body condition, was correlated with our participants’ navigation performance. These data link self-centered processes during spatial navigation to entorhinal and retrosplenial activity.

We report that enhanced self-related processing, induced by sensorimotor congruency between our participants’ physical body and the seen corresponding avatar during spatial navigation improved spatial navigation performance. Participants committed smaller distance errors and navigated in shorter paths when seeing the avatar associated with synchronous sensorimotor stimulation (Fig. 2c, d). Previous work has revealed that modulations of BSC not only alter body- and self-related processes^39,56,57^, but also affect spatial representation and episodic memory^24,27,58,59^. The present data extend BSC to spatial navigation.

In addition to overall improved spatial navigation performance in the BSC condition, the present data also reveal that the subjective BSC changes (measured by questionnaires) were associated with a behavioral BSC change characterized by a drift in self-location. Although participants navigated more precisely in the Body condition (smaller distance error), they stopped farther away from the arena’s border when navigating with a self-identified avatar (as compared to the No-body condition), revealing a BSC change that altered where our participants located themselves with respect to external landmarks. In the previous BSC literature, bodily stimulation has been shown to induce a positional recalibration of self-location, referred to as drift in self-location^18^ (for review, see Dieguez & Lopez^60^). Building on this literature, we suggest that the navigation-related effect of greater distance from the border in the BSC-inducing body condition might relate to the drift in self-location. A link between the drift effect and BSC is further corroborated by the significant correlation between the BSC questionnaire ratings (Q2) and the magnitude of the distance from the border. Accordingly, we suggest that these self-location findings extend previous BSC data using gait responses^18,39^, or mental imagery tasks^19,21,40^ to the field of spatial navigation (for review, see refs. ^21,40,60,61^) and confirm our hypothesis that experimentally-induced alterations of the sense of self affect processes of spatial navigation. The present self-location and spatial navigation data suggest that the reference point during spatial navigation in the Body condition was processed with respect to the avatar in the VR space, linking enhanced BSC and sensorimotor processing centered on the avatar with improved spatial navigation performance.

Entorhinal grid cell activity in animals and humans has been consistently shown to depend on self-motion-related cues, originating from the individual’s body, as well as sensory cues from the environment^1,2,5,6,11^. Prior human studies have also observed GCLR linked to various cognitive functions related to spatial representation^30,33–35,62,63^. However, it is not known how GCLR in humans depends on self-centered processes related to BSC and self-identification with a body in particular. In the present study, we provide fresh insights into this relationship. Interestingly, contrary to our initial prediction, we observed a decrease of GCLR in EC in the Body condition, while the typical hexadirectional modulation in EC was observed during the condition that was similar to previous spatial navigation studies (i.e., the No-body condition)^30–32^. Accordingly, the present data link GCLR reduction to enhanced self-centered processing characterized by enhanced self-identification with an avatar.

We expected enhanced entorhinal GCLR, not a GCLR decrease, for the condition with enhanced self-centered processing during navigation with a self-identified avatar, but observed a GCLR decrease. We propose that (1) conflicts between different spatial reference frames and (2) decoupling of navigation and GCLR as non-exclusive mechanisms have led to the decreased entorhinal GCLR. First, distortions of grid cells’ spatial representation have also been linked to the integration of incongruent visual and self-motion cues in rodents^11^, showing that grid cell firing field maps can be elongated or shrunk by the relationship of proprioceptive versus visual cues and that the distorted grid patterns are no longer sixfold symmetric. Similarly, the GCLR decrease might be due to conflicts between spatial information that is referenced to different reference frames in the Body condition (i.e., visual environmental cues referenced to the avatar vs. vestibular or proprioceptive cues anchored to the physical body). The present changes in self-identification were induced by fully-controlled sensorimotor stimulation that linked the physical body with the virtual avatar. It is thus possible that such stimulations may have interfered with the integration across different bodily reference frames during navigation (i.e., visual environment-avatar versus participant’s body in the scanner). Unlike during standard virtual navigation (i.e., No-body condition), body-derived cues (e.g., proprioceptive, vestibular signals) in the Body condition that are referenced to one’s physical body could be in conflict with those falsely integrated with visual cues referenced to the virtual avatar thereby leading to the observed decrease of GCLR in EC. Of note, distinguished from the grid cells in rat EC^1^, the grid pattern in mice was disrupted in the absence of visual input^64^, suggesting different levels of reliance on visual cues across species. Therefore, in humans who rely more on visual input^65,66^, visual input may be sufficient to generate GCLR^28,30^, but increased reliance on additional bodily cues that conflict with the visual cues could disrupt the hexadirectional modulation in the Body condition. Second, the decreased GCLR observed in the Body condition could be a consequence of a decoupling between the spatial navigation behavior and the grid cell system, reflecting the flexibility of its involvement in the spatial representation due to enhanced egocentric processing^31,32^. Indeed, a recent human study showed that theta oscillations in the medial temporal lobe encoding the boundary information were not present when the navigation was not relevant to the target search task^67^, suggesting the dynamic involvement of the sub-systems in the medial temporal lobe. More relevant to our study, it has been also reported that spatial navigation based on a non-allocentric strategy does not recruit the hippocampal-entorhinal regions, while activation of the area is observed during navigation based on allocentric strategies^68^. Therefore, we suggest that the reduction of GCLR in the Body condition could be related to comparable mechanisms: enhanced self-centered processing in a state of enhanced self-identification with the avatar could boost egocentric processing and indirectly decrease the reliance on allocentric processing. This is also consistent with our RSC and IPS data which are discussed further below. The suggested decoupling between GCLR and spatial navigation in the Body condition could also explain the observed improvement in spatial navigation performance despite GCLR reduction that seems to contradict the known role of grid cells in spatial navigation^5,41,69^. Compatible with this suggestion, we did not detect a correlation between GCLR and spatial memory performance, as was also not the case in many previous human grid cell studies using similar spatial navigation paradigms^30,32,34,70^. Such a correlation, however, was observed for RSC, a key region in spatial navigation that has been shown to mediate ego- and allocentric processes.

Other factors may have led to a GCLR decrease. However, further results from the present study suggest that the decreased GCLR is not attributable to navigation-related factors (such as speed, central navigational preference, differences in target locations, training, navigated trace length, and drift in self-location) nor to head-motion artifacts (see Supplementary Notes). It could be argued that the reduced GCLR during the Body condition might be related to distraction or visual occlusion of the VR scene by the avatar. However, as shown by the exemplary task scenes (Fig. 1; see also Supplementary Movie 1), only a relatively small part of the visual field was occluded by the avatar. Importantly, distraction or occlusion (due to the avatar) should lead to decreases in spatial navigation performance and we observed the opposite effect: spatial navigation performance was better in the Body condition. Also, there was only minimal spatial information that participants could acquire from the lower part of the scene (occluded by the avatar) as all landmarks were placed in a distal position and not in the task arena, where they were navigating. In addition, we note that previous human grid cell studies with strong GCLR responses have used avatars during navigation: Jacobs et al. (2013) and Maidenbaum et al. (2018) that also partially occluded the scene (i.e., avatar hands, holder)^31,71^, suggesting that visual occlusion or changes in optic flow due to the avatar cannot account for the decreased GCLR in the present study. It is, therefore, unlikely that the present effects were caused by scene occlusion due to the avatar. However, as we cannot fully exclude that differences related to visual input, due to the avatar, might have contributed to the lower GCLR in the Body condition, future work could evaluate GCLR as well as behavioral performances in an object control condition, as has been done in prior BSC research^18^.

EC is not the sole brain region that determines spatial navigation performance. For example, Kunz et al. (2015) reported that compensatory mechanisms in the hippocampus account for GCLR reduction despite maintained spatial navigation performance^31^. Spatial navigation is based on activity within a large distributed network, involving IPS/PPC, RSC, and several other regions^44–46,48,72–74^, also supported by the whole-brain analysis in the present study revealing activations in bilateral retrosplenial cortex (RSC), bilateral parahippocampal gyrus and right lingual gyrus^42–45^. From these regions, only RSC activity was significantly enhanced in the Body condition, where we also observed reduced GCLR. Moreover, we further associated higher RSC activation with improved spatial navigation performance (i.e., smaller distance error). These data are compatible with a compensatory role of RSC activity for the decreased GCLR in EC. This is in line with the recent work by Bierbrauer et al. (2020), which suggests that RSC is involved in compensatory navigational mechanisms when GCLR is affected (i.e., in a genetic risk group for Alzheimer’s disease)^70^. Thus, in the present study, increased right RSC activity in the BSC condition may reflect compensatory mechanisms for decreases in entorhinal GCLR.

Thus, the attenuation of entorhinal GCLR could also be related to decreased reliance on allocentric spatial processing associated with the boosted egocentric processing by the BSC modulation, further corroborated by increased activity in posterior parietal regions (i.e., IPS, RSC) associated with the decreased GCLR (see Fig. 4). In support of our proposal, previous work consistently linked human RSC activation to spatial navigation^45,47,54,55,75–77^ and RSC has been proposed to be important for orienting to landmarks^70^, a central feature of our experimental design as distal landmarks only provided orientation cues. Moreover, clinical research consistently linked human RSC damage (especially of right RSC) to orientation impairments in spatial navigation^47,78,79^. Hence, the correlation we observed between RSC activation and spatial memory precision (i.e., distance error) extends previous findings on the role of RSC in spatial navigation and adds the important finding that spatial navigation-related activity in RSC, depends on the level of BSC. RSC has been regarded as a mediator between self-centered (egocentric) and environmental (allocentric) processes in PPC and medial EC, respectively^54,55^. RSC integrates body-derived self-motion cues while mapping one’s location in the environment^80,81^. Moreover, RSC has also prominently been associated with several self-related cognitive processes beyond spatial navigation, such as self-orientation in time^82^, across social dimensions^83^, integration of self-referential stimuli^84^, autobiographical memory^85,86^, and BSC^15,49,50^.

Finally, the present IPS data further extend the BSC-related changes we observed in RSC and EC. IPS, and more generally PPC, is regarded as a core region for egocentric spatial representation in humans^46,52,87,88^. Supporting this human work, rodent studies reported that neurons in PPC encode self-centered cues independent of the external environment (e.g., self-motion and acceleration) during navigation^89,90^. In addition, many human BSC studies reported IPS activation when key components of BSC (e.g., self-location and self-identification) were modulated by multisensory stimuli, and IPS is considered a key BSC region^14,15,49–51^. Reporting increased IPS activity associated with solid behavioral evidence of drift in self-location during spatial navigation when our participants navigated with a self-identified avatar, these IPS and RSC data extend previous BSC neuroimaging findings to the field of spatial navigation. In the Body condition, we observed increased IPS activation that has been linked with BSC and egocentric spatial processing. Many previous studies showed that body-referenced cues (e.g., vestibular, motor, and proprioceptive signals) are processed in PPC and provide crucial inputs to grid cells^10,11,28,91^. We argue that the present data link BSC-related processing as manipulated by online sensorimotor stimulation to egocentric processes in IPS and to allocentric grid cells in EC, suggesting that human grid cell-like activity in EC reflects ego- and allocentric processing demands. Enhanced RSC activity in the Body condition and the mediating role of RSC between ego- and allocentric processes in spatial navigation, as well as between PPC and EC^54,55^, is compatible with this suggestion. Accordingly, we propose that the BSC changes, characterized by strengthened self-centered processing referenced to the avatar, associate enhanced egocentric, self-centered, processing in IPS, with altered ego- and allocentric processing in RSC, and with reduced allocentric spatial representation, GCLR in EC.

The present results associate enhanced spatial navigation performance with decreased GCLR in EC when participants were navigating during an enhanced BSC state (i.e., navigating with a self-identified avatar), showing that enhanced self-centered bodily processing boosted navigation behavior and decreased entorhinal GCLR. These decreases in entorhinal GCLR were associated with increases in RSC activity, another major spatial navigation region that has been shown to mediate between ego- and allocentric spatial representation. Moreover, RSC activity, stronger during the Body condition, was correlated with our participants’ navigation performance. These data link the sense of self behaviorally and neurally to spatial navigation and opposing activations in the entorhinal and retrosplenial cortex.

## Methods

### Participants

Twenty-seven healthy right-handed participants (13 males and 14 females; mean age 25.3 ± 1.96) with normal or corrected-to-normal vision were recruited from the general population. The number of participants, 27, was chosen according to the minimum sample size calculated from Nau et al. (2018) to reproduce conventional grid cell-like representation. Participants were naive to the purpose of the study, gave informed consent in accordance with the institutional guidelines (IRB #: GE 15-273) and the Declaration of Helsinki (2013), and received monetary compensation (CHF20/hour). Two participants who entered random answers to the questionnaire were excluded from the questionnaire analysis (i.e., both pressed the response button repeatedly at incorrect moments during the experiment; further confirmed by post-experiment debriefing). A participant whose structural image was examined as abnormal by a medical investigator was excluded from the fMRI analysis. Another participant was excluded from the grid cell-like representation analyses due to severe image distortions and signal drop in EC (~2.3% of voxels in the EC were above the global average of mean EPI). A session with head drift greater than 3 mm was also excluded from fMRI analyses.

### MRI-compatible virtual reality (VR) spatial navigation task

Stereoscopic visual stimuli were provided via MRI-compatible goggles (VisualSystem, Nordic NeuroLab: 800 × 600 resolution, 40 Hz refresh rate). An MRI-compatible joystick (Tethyx, Current Designs) was used to perform the task inside the MRI scanner. The task program including the virtual arena and virtual objects was implemented with Unity Engine (Unity Technologies, https://unity3d.com).

The task arena did not contain any landmark inside, and distal landmarks providing orientation cues were placed outside of its boundary. The task procedures were adopted from previous human spatial navigation studies (Fig. 1a)^30,31,34^. Each session of the task started with an encoding phase, in which participants had to sequentially and repeatedly memorize the locations of three objects (at least three times per object), while freely navigating inside the circular arena using a joystick. In each trial, following encoding, participants were asked to recall and return to where a cued object was. (1) During the Cue phase, one target object among the three encoded objects was represented as floating in the virtual scene for 2 s. (2) After the cue disappeared, they had to navigate to the target location, where they recalled the target object was placed before, by manipulating the joystick. After reaching the recalled position, they pressed a button on the joystick to confirm their response (i.e., Retrieval phase). (3) Sequentially, participants were asked to report the distance error they estimated to have committed (the distance between the reported and correct object’s location; Fig. 1a-top-right) by indicating on a continuous scale ranging from 0 to 110 vm (i.e., Distance-estimation phase). (4) Following the distance estimation, the object appeared at its correct position, and participants had to navigate and collect the object (i.e., Collection phase) before starting the next trial. The Collection phase was to provide a participant with an additional encoding cue (i.e., feedback), but also to ensure that the spatial traces spanned various directions designed to allow the analysis of grid cell-like representation (GCLR) (Fig. 2b)^34^. At the end of each session, participants were presented with a virtual knife directed toward them, in order to measure how threatened they felt as a subjective measure of BSC change (Fig. 2a-Q2; see Questionnaire section). During the task, locations of participants in the virtual arena were recorded with respect to the first-person viewpoint (regardless of the avatar), identically across the conditions.

In total, the experiment consisted of six sessions divided into three blocks, aimed at comparing the two conditions (Fig. 1b) in terms of both brain activity and spatial navigation performances. The order of the conditions was pseudo-randomized within each block and counterbalanced between participants (N-B/B-N/N-B or B-N/N-B/B-N). As the task was self-paced, the duration of each session varied depending on the participant’s performance (mean round duration: 9.0 ± 0.70 min), but didn’t differ between the conditions (*t*-test: *p* = 0.92).

### BSC modulation with the virtual body

The Body condition with a neutral body-shaped avatar was designed to experimentally modulate BSC, more specifically, to enhance self-identification with the virtual avatar in the VR environment compared to the baseline: the No-body condition. The avatar was designed to be gender-neutral and gray-skinned without hair. The virtual body was in the same posture as a participant—supine—and its right hand was shown as congruent with respect to the participant’s hand movements controlling the joystick. These settings of avatar were chosen to achieve sensorimotor congruency between the participant’s body and the avatar, which has been reported to lead to self-identification with the avatar^37,38,92,93^.

### Questionnaire

At the end of each session, participants were asked to answer four questions using a Likert scale ranging from −3 (strongly disagree) to 3 (strongly agree). The questions were randomly ordered across sessions and participants answered with the joystick. Q1 (“I felt as if what I saw in the middle of the scene was my body”) was intended to measure self-identification with the avatar. Q2 (“I felt as if the threat (knife) was toward me”) was also designed to measure the degree of threat towards the participant. Q3 (“I felt dizzy”) sought to measure cybersickness (Supplementary Fig. 1a). Q4 (“I felt as if I had three bodies”) served as a general control question. A short debriefing was carried out after participants had completed the experiment.

### Prescreening and training in the Mock scanner

The participants were trained to perform the spatial navigation task in a mock scanner. The training consisted of one session of the No-body condition and lasted around 10 min, keeping them naive to the experimental condition. To avoid potential carryover effects, we used different virtual objects and environments than those used in the main experiment. This training also allowed us to exclude participants experiencing a severe cybersickness caused by navigation in the VR environment^94^.

### MRI data acquisition

MRI data were acquired at the Human Neuroscience Platform of the Campus Biotech (Geneva, Switzerland), with a 3 T MRI scanner (SIEMENS, MAGNETOM Prisma) equipped with a 64-channel head-and-neck coil. The task-related functional images covering the entire brain were acquired with a T2*-weighted Echo Planar Imaging (EPI) sequence with the following parameters: TR = 1000 ms, TE = 32 ms, Slice thickness = 2 mm (no gap), In-plane resolution = 2 mm × 2 mm, Number of slices = 66, Multiband factor = 6, FoV = 225 mm, Flip angle = 50˚, slice acquisition order = interleaved. The structural image per participant was recorded with a T1-weighted MPRAGE sequence with the following parameters: TR = 2300 ms, TE = 2.25 ms, TI = 900 ms, Slice thickness = 1 mm, In-plane resolution = 1 mm × 1 mm, Number of slices = 208, FoV = 256 mm, Flip angle = 8˚. In addition to that, B0 field map (magnitude and phase information, respectively) was acquired to correct EPI distortion by inhomogeneous magnetic fields (especially, near the medial temporal lobe).

### fMRI data preprocessing

MRI data were preprocessed with SPM12 (http://www.fil.ion.ucl.ac.uk/spm). Functional images were slice-time corrected, realigned, unwarped using a B0 field map, and coregistered with the individual T1-weighted structural image. For the conventional generalized linear model(GLM) analysis (e.g., region-of-interest (ROI) analysis of task-related regions and IPS), the images were normalized to Montreal Neurological Institute (MNI) space, to allow a second-level GLM analysis designed to localize commonly activated brain regions across participants. Following previous studies, other analyses regarding grid cell-like hexadirectional modulation with the EC as a main ROI were conducted in the native space without normalization to avoid additional signal distortion^31,32^. All preprocessed functional images were smoothed with a 5 mm full-width-half-maximum Gaussian smoothing kernel as the final preprocessing step.

### Definition of the EC ROI in participants’ native space

Participant-wise EC ROIs for the analysis of grid cell-like representations(GCLR) were defined using Freesurfer (v6.0.0, http://surfer.nmr.mgh.harvard.edu) as described in previous studies^31,95^. Briefly, a cortical parcellation was automatically conducted by the software with the individual T1 structural images based on the Desikan-Killiany Atlas^96^. The bilateral EC labels generated from the parcellation were taken as an individual ROI and were examined manually by overlapping them on the corresponding structural image. Subsequently, the ROIs in the “freesurfer conformed space” were transformed into volume ROIs in the participant’s native space and, then, coregistered and resliced to the mean EPI images.

### Analysis of grid cell-like representation (GCLR)

The Grid Code Analysis Toolbox (GridCAT v1.03, https://www.nitrc.org/projects/gridcat) under MATLAB 2018b (The Mathworks) was used to analyze GCLR^97^, following a seminal method which was proposed by Doeller et al. (2010). The analysis was comprised of two steps with mutually exclusive datasets. As a first step, with one of the partitioned datasets, a first GLM (GLM 1) was used to calculate ${\beta }_{1}$ and ${\beta }_{2}$, using two parametric modulation regressors: ${\cos }\left(6{\theta }_{t}\right)$ and ${\sin }(6{\theta }_{t})$ respectively, where ${\theta }_{t}$ is heading direction during the navigation in time(t). Then, using the betas, voxel-wise amplitude($A$) and grid orientations($\varphi$) were respectively estimated by

1 $$A=\sqrt{{\beta }_{1}^{\,2}+{\beta }_{2}^{\,2}}$$

2 $$\varphi ={{\tan }}^{-1}({\beta }_{2}/{\beta }_{1})/6$$

In the second part of the analysis, a putative grid orientation was calculated by the weighted average of the voxel-wise grid orientations($\varphi$) in the ROI (i.e., EC) with the voxel-wise amplitude($A$) of each voxel as its weight. Subsequently, based on the putative grid orientation($\varphi$) and moving direction information(${\theta }_{t}$), a second GLM (GLM2) estimates an amplitude of GCLR in the EC, selectively (1) by contrasting regressors for navigation toward grid-aligned ($\varphi$ + 0,60,120,…,300) vs. misaligned ($\varphi$ + 30,90,150,…,330) direction or (2) by applying a sixfold symmetric sinusoidal parametric modulation regressor:

3 $${\cos }\left(6\left({\theta }_{t}-\varphi \right)\right)$$

We calculated GCLRs with both methods respectively, to confirm that our result does not depend on either of the methods. In order to optimally utilize all available data and improve the signal-to-noise ratio, a cross-validation method with multiple partitions was adopted^35^, instead of dividing the data into two equal halves. An amplitude of sixfold representation for a given session was calculated based on the grid orientation estimated with the other five sessions and the process was repeated for every session. The session-wise results were summed together to estimate the overall grid cell-like representation of the participant.

### Calculation of condition-wise GCLR

The classical GCLR in the previous step was calculated with all six sessions without taking into account the experimental condition of each session, which implies that each estimate does not purely represent a magnitude of the hexadirectional modulation in the corresponding experimental condition. Besides, as two different experimental conditions were timely intermingled, grid orientations across sessions even within the same condition could be unstable, which critically affects the GCLR estimation^31,32^. Hence, we calculated session-wise BOLD contrasts between aligned vs. misaligned movement independently from the other sessions, using data from the single session only. Leave-one-out cross-validation with ten partitions was performed for each session to optimally utilize the dataset and maximize the signal-to-noise ratio (See the Methods section above)^35^. In more detail, each session was divided into multiple three-second (3 s) bins. The first out of each of these ten bins became the first partition (where the bin index modulo 10 equals 1) and each second bin from every ten bins became the second partition (bin index modulo 10 equals 2), and so forth until the tenth partition. Notably, this method was dedicated to the comparison of GCLR between the Body and No-body condition, rather than the demonstration of hexadirectional modulation in contrast to the controls (e.g., four/five/seven-fold symmetry) which was already fulfilled. Calculated session-wise results were averaged by condition to get the condition-wise GCLR, which is robust to potential temporal instability of grid orientations across sessions.

### Temporal and spatial stabilities of grid orientations

Spatial stability was defined as the homogeneity of voxel-wise grid orientations within EC. To assess the spatial stability of a session, Rayleigh’s test for non-uniformity of circular data was calculated with the voxel-wise grid orientations within EC. Rayleigh’s *z*-value was taken as an index of the spatial stability of the session^31,32^. Temporal stability was defined as the stability of grid orientations over time. For each session, it was computed by the circular standard deviation of ten grid orientations estimated during each of the ten cross-validations of GCLR described above. Of note, the ten grid orientations were calculated with ten different data portions from different time points. Therefore temporally stable grid orientations should remain similar across folds, resulting in a small standard deviation (Supplementary Note 1).

### Generalized linear model (GLM) analysis to detect task-related brain regions

Whole-brain GLM analysis using normalized functional images was performed to detect brain regions, possibly accounting for changes in spatial navigation performance. Beta values during the task phase were extracted using GLM analysis with the tailored regressors using SPM12 (Supplementary Table 1). First, with the parametric modulation regressor, we searched for brain regions where its activation during the ‘Retrieval’ phase was correlated with the “distance error”. However, this analysis did not reveal any significant clusters after the voxel-level family-wise error correction (FWE). Second, we assessed contrasts (Body > No-body) during the task phases before participants finished the retrieval procedure (i.e., Cue + Retrieval), as such responses could be responsible for the spatial navigation performance of the trial. However, again, no cluster (extent threshold >20 voxels) survived after voxel-level FWE correction (*p* < 0.05).

### ROI analysis of task-related BOLD activity by using the functional localizer

Next, we performed ROI analysis to investigate brain regions involved in spatial navigation performance. Functional ROIs relevant to the spatial navigation task were defined by the functional contrast (Task: Cue + Retrieval + Feedback + Collection > implicit baseline; i.e., orthogonal to the conditions of interest: the Body and No-body) in second-level GLM using a voxel-level threshold family-wise error-corrected for multiple comparisons of *p* < 0.05 and using an extent threshold of 20 voxels (Fig. 4a; Supplementary Fig. 5; and Supplementary Table 2). In order to search for task-related activity accounting for improved spatial navigation, mean beta values within the ROIs during the task phases before reaching the recalled location (i.e., Cue + Retrieval) were compared between the two experimental conditions.

### ROI analysis of intraparietal sulcus region (IPS)

We anatomically defined the ROI for the bilateral IPS based on the normalized functional images (in MNI space) by using SPM Anatomy Toolbox based on the Jülich probabilistic cytoarchitectonic maps^98^. Betas during the Retrieval phase in IPS were compared between the Body and No-body conditions.

### Statistics and reproducibility

Statistical assessments for behavioral and fMRI data were performed with R (v3.5.3 for Windows, https://www.r-project.org/) and RStudio (v1.2.1335, http://www.rstudio.com). Outlying data points outside of the standard deviation range, −3 to 3, were excluded from the statistical analysis. For the behavioral parameters having a value per trial (distance errors, navigation trace length and time, distance from the border), mixed-effects regressions (lme4_1.1-18-1, a package of R), which include condition as a fixed effect and random intercepts for individual participants, were used to assess statistical significance. Random slopes were included as far as there were no estimation failures. For the other parameters with no single-trial estimates (e.g., questionnaire ratings, spatial/temporal stability of the grid orientation, task-related BOLD activity), a non-parametric two-sided Wilcoxon signed-rank test was used. The conventional sixfold patterns of GCLR were assessed with a one-sided Wilcoxon signed-rank test, as expected from previous work^30,35^. However, the condition-wise comparison of the GCLRs was performed with a two-sided test. To indicate the effect size of the Wilcoxon signed-rank test, *r* values were calculated with the following formula:

4 $${{{{{\rm{r}}}}}}={{{{{\rm{Z}}}}}}/\sqrt{N}$$

Assessments of correlations were conducted using mixed-effect regression models so that the condition-induced effects within participants are properly taken into account by the random effect of a participant, in addition to the across-participants effect. In order to assume the best-fit distribution and apply proper parameters for each mixed-effects regression used, data distribution of each dependent variable was assessed using fitdistrplus(v1.0-11, a package of R).

### Reporting summary

Further information on research design is available in the Nature Research Reporting Summary linked to this article.

## Supplementary information

Supplementary Information

Description of Supplementary Information

Supplementary Movie 1

Reporting Summary

## Data Availability

The data underlying the results and the main figures of this study have been uploaded on the open science framework (10.17605/OSF.IO/U8VHA) and are open to the public.

## Code availability

The customized codes that were used for this study are available on the open science framework (10.17605/OSF.IO/U8VHA) and open to the public.
