# Supplementary material for: Sense of self impacts spatial navigation and hexadirectional coding in human entorhinal cortex
Source: Commun Biol. 2022 May 2;5:406. doi: 10.1038/s42003-022-03361-5 (PMC9061856; doi:10.1038/s42003-022-03361-5)
Supplement: Supplementary file 1 — Supplementary Information [file 42003_2022_3361_MOESM1_ESM.pdf]

Supplementary Information

Supplementary Notes

Supplementary Note 1: Detailed analysis on the subparameters of GCLR

We further assessed the subparameters of GCLR (i.e., voxel-wise amplitude, temporal/spatial stability of the grid orientation) to better understand the mechanisms underlying the decreased GCLR we observed in the Body condition. We averaged voxel-wise amplitudes of the estimated six-fold sinusoidal curve in the EC region of interest (ROI) regardless of the grid-orientation value the voxel has. The voxel-wise amplitudes were calculated to estimate mean grid-orientation during the conventional GCLR analysis, and have been regarded to reflect grid cell-like activity at the voxel level (i.e., the values are used as weighting factors while calculating the mean grid orientation in the ROI) [1,2]. Comparing the mean voxel-wise amplitudes across conditions, we replicated our finding that the values were significantly lower in the Body vs. No-body condition ($Z = 2.68$, $r = 0.54$, $p = 7.37e-03$, $n = 25$; Supplementary Figure 4a). We also found a significant correlation between the mean voxel-wise amplitudes of the hexadirectional modulation and the condition-wise GCLRs (linear mixed-effects regression; NumDF = 1, DenDF = 142, $F = 4.58$, $p = 0.034$; Supplementary Figure 4d). Notably, the values were even more strongly correlated to the absolute values of the GCLR (NumDF = 1, DenDF = 135.9, $F = 20.69$, $p = 2.22e-03$; Supplementary Figure 4g), consistent with the theoretical expectation that a sign of GCLR can be inverted to negative by wrongly estimated grid cell orientation, although the specific BOLD modulation itself is prominent. The spatial stability of grid orientations, defined as the homogeneity of voxel-wise grid orientations within the EC-ROI, was quantified by Rayleigh's Z (see Methods) [3,4]. In addition, to quantify temporal stabilities of the grid orientations during a session, standard deviations of grid orientations estimated from different portions of the session were calculated (indexing instability rather than stability; see Method). These analyses revealed that both spatial and temporal stability of grid orientations were not found to significantly differ between the two conditions (Spatial stability: $Z = 1.25$, $r = 0.25$, $p = 0.210$; Temporal instability: $Z = 1.47$, $r = 0.29$, $p = 0.141$; Supplementary Figure 4b-c). We note, further, that both spatial and temporal stability, significantly influenced the condition-wise GCLRs (NumDF = 1, DenDF = 142, Spatial stability: $F = 9.27$, $p = 2.78e-03$; Temporal instability: $F = 23.81$, $p = 2.82e-06$; Supplementary Figure 4e-f).

Supplementary Note 2: Assessment of GCLR in RSC and IPS

In an additional analysis, we further assessed whether the reduced GCLR in the Body condition was associated with greater engagement of GCLR in RSC or IPS. However, no significant GCLR (greater than 0) nor a significant difference between conditions was detected in either region (see Supplementary Figure 6).

Supplementary Note 3: Additional control analyses for the potential impact of differences in behavioral parameters on GCLR: correlation and re-sampling

We performed further in-depth analyses to assess a possible influence of behavioral parameters that may have differed between Body and No-Body conditions (distance error, navigated trace length, distance to the border) and how that may have influenced GCLR activation. First, we assessed the correlation between GCLR and each of the parameters (separately) and found no significant relationship between any of these parameters (distance error: $p = 0.52$, navigated trace length: $p = 0.32$, distance to the border: $p = 0.48$), suggesting that GCLR was not related to any of these behavioral parameters and further corroborating that the reduced GCLR in the Body-condition was not merely a consequence of the difference in these parameters.

In addition, to provide even further evidence, we re-calculated GCLR by selectively sampling the data to compensate for the difference in the behavioral parameters: as a consequence, the difference in those parameters became statistically non-significant (border distance) or even led to a reversal of the difference between two conditions (distance error, path distance). For example, in the case of distance error, we excluded the round with the best performance among all Body condition rounds while excluding the worst round among No-body conditions for each participant. As a result, their performance became significantly worse in the Body condition in the sampled dataset ($p = 2.50e-4$, $r =0.73$). However, the respective GCLR was still non-significant in the Body condition ($p = 0.23$, $r = 0.24$), while it remains prominent in the No-body condition ($p = 1.4$ e-3, $r = 0.64$). The same procedure was repeated for the rest of the parameters in a way to compensate or reverse the condition-wise behavioral differences (resampling-border distance: $p = 0.731$, $r = 0.069$; -navigated trace length: $p = 1.03e-4$, $r = 0.78$; see the left panels of Supplementary Figure 8). However, any of the samplings led to neither significant GCLR in the Body condition (resampling border distance: $p = 0.18$, $r = 0.27$; -navigated trace length: $p = 0.12$, $r = 0.31$) nor the non-significant GCLR in the No-body condition (resampling-border distance: $p = 2.1e-3$, $r =$

0.62;-navigated trace length: p = 7.4e-3, r = 0.54), strongly corroborating that the GCLR results we observed in the current study did not originate from the difference in those parameters.

Of note, while we again confirm with this analysis that the mean of GCLR in the Body condition was lower than the mean GCLR of the No-body condition for all three resamplings, the difference was statistically significant only for dMoved (navigated trace length) resampling (p = 0.039). These results might be due to 1) influence of behavioral differences or 2) decrease of statistical power because of the exclusion of 1/3 of the dataset. In order to assess this further, we again resampled the dataset but this time in the opposite way (i.e., to exaggerate the differences in the behavioral parameters). The second resampling again showed that GCLR in the Body condition was not significant, while GCLR of the No-body condition was significant (Supplementary Figure 9). More importantly, the statistical difference between the GCLRs in the Body and No-body condition was the same as for the first resampling: the difference was only significant for the dMoved resampling (p = 0.034). These results strongly suggest that 1) condition-wise difference in GCLR is very unlikely due to the difference in those behavioral parameters, and 2) the non-significant differences in the resampling analyses could be derived from the decrease in statistical power.

Supplementary Note 4: GCLR and subparameters

By nature, the spatial/temporal stability of the grid orientation and the voxel-wise amplitude of the hexadirectional modulation are deeply intermingled in the estimation of GCLR. However, our in-depth analyses, to some extent, dissociated each factor (albeit not completely). For instance, if different grid cell modules encode distinct grid code, it will lead to decreased spatial stability but intact voxel-wise amplitude, which was not the case in our results.

Supplementary Note 5: Navigation speed

Rodent single neuron recordings and human GCLR results have demonstrated that grid cell activity depends on the navigation speed of the subject[1,5,6]. However, speed differences cannot account for the present GCLR reduction, because navigation velocity was fixed not to differ between both conditions. Moreover, the average moving speeds of each trial (navigated distance/time) also did not differ between conditions (p = 0.46). We further assessed the relationship between the average navigation speed and the GCLR while also taking into account the effect of the experimental condition in the linear mixed-effect model. These results show that

the GCLR reduction is not related to the difference in navigation speed (effect of the average navigation speed on GCLR was not significant, p = 0.54).

Supplementary Note 6: Egocentric vs. Allocentric strategies during the task

As argued in the main text, we contend that the vanished GCLR could be derived from the decreased reliance on allocentric spatial processing associated with the boosted self-centered processing by the BSC modulation. However, we failed to observe any explicit behavioral differences suggesting a change of our participants' navigation strategies during the task, which also possibly impacts GCLR by the corresponding involvement of allocentric GCLR processing. The behavioral parameters that possibly evidence the changes in the strategies did not significantly differ across conditions (e.g., central preference as reported in Kunz et al. 2015, or perpendicular angles to the border that may reflect their tendency to match the view of the background scenery). As suggested by previous human navigation literature[7-9], enhanced self-centered processes (e.g., additional body-derived cues) necessarily lead to significant changes in neither navigation strategy (e.g., the influence of body-based cues) nor spatial navigation network in the brain. Rather, their change could be more related to the type of the task and scale of the environment. Although we did not directly investigate experimentally how activity in RSC and EC depends on ego- and allocentric navigation strategy, we speculate that the enhanced self-centered processing was related to a shift towards egocentric navigation in the Body condition based on brain activity changes we observed, unlike the study of Huffman and Ekstrom (2019). Future work should assess the impact of explicit manipulations of navigation strategy (ego- vs. allocentric) on GCLR and other nodes of the spatial navigation network (e.g. RSC).

Supplementary Note 7: Participant's position

Humans rarely navigate in a supine position and it could be argued that the decreased GCLR we observed is related to such mechanisms, potentially enhanced by showing a supine avatar during navigation. However, several arguments speak against this hypothesis. First, the participants were in a supine position in both conditions. Second, this was the same position as tested in all previous human GCLR work using fMRI. Third, as previous spatial navigation work did not quantify experienced self-location and self-identification with the avatar, the experienced position of participants for previous GCLR data is, therefore, to the best of our knowledge, not known. We

believe that although humans mostly navigate in an upright position, especially the sitting position (e.g. car, wheelchair) and also the supine position (e.g. luge) are suitable positions to test spatial navigation. More work is necessary for this important issue. The present data suggest that for any spatial navigation paradigm (upright, sitting, supine) the control of the different bodily reference frames (i.e. somatosensory, visual), as well as the quantification of the subjective bodily reference frame (i.e. self-identification, self-location), should be controlled.

Supplementary Note 8: Drift in self-location

It could be also interesting to assess whether the drift in experienced self-location that we observed in the Body condition (Fig. 3) affected the grid cell pattern (e.g., shift of hexagonal grid). However, our GCLR analyses were based on the seminal work by Doeller and colleagues (2010), who based GCLR on the analysis of the heading direction-dependent hexadirectional BOLD modulation, which is calculated with heading direction information that is independent of the individual's location in the arena. Hence, as the small drift in self-location (~1vm) that we report was along the same heading direction, there can be no change in GCLR (i.e., a heading-direction-dependent BOLD modulation). Further dedicated experiments would be required, inducing drifts in self-location, that possibly generate or affect detectable GCLR metrics.

Supplementary Note 9: GCLR and spatial memory performance

While some GCLR studies reported a positive correlation between GCLR and spatial memory performance[1,10], different parameters and neuroimaging modalities were used for these correlations. Importantly, not all studies used the same spatial navigation paradigm, which may greatly affect the involvement of grid cell system (e.g., pre-training, ego- vs. allocentric strategy). Hence, it is difficult to generalize from the results of these different studies to our results.

Supplementary Note 10: GCLR and self-identification

One might argue that enhanced self-identification with the navigating agent in VR (i.e., Body condition) should enhance GCLR, contrary to our results. However, it has been shown that place cells also encode the locations of others[11,12] and this may also apply to grid cells as tested here for GCLR with an avatar that we showed in a virtual environment. Human GCLR has also been

reported during conceptual spatial mapping[13], and thus manipulations not related to the position of the navigating agent per se. Accordingly, one cannot directly assume that stronger self-identification with respect to an avatar in a virtual environment leads to enhanced GCLR.

Supplementary Note 11: Deformation of grids due to physical restrictions in the navigation arena

It is also possible that the absence of GCLR in the Body condition originated from the deformation of grid cells' grids, as the GCLR is calculated on the premise of the six-fold symmetric grid firing fields map. For instance, in line with rodent data[14], physical restrictions of navigation possibilities in the environment (e.g. by physical spatial constraints) have been shown to disrupt the hexadirectional GCLR modulation in humans[15]. In the presence of such restrictions, firing fields of grid cells do not tile in typical hexagonal grids, reflecting a change of navigational processing corresponding to the dimensions of the navigation corridor. However, we do not think that deformation-related mechanisms account for the observed GCLR decrease in EC, because the two conditions were performed in the same virtual arena. Although, the drift in self-location that we observed in the Body-condition may relate to such deformation-related mechanisms, a perceived physical restriction, but our study was not designed to test this explicitely and entorhinal GCLR did not reflect drift in self-location.

Supplementary Note 12: Effect of time on the BSC modulation and GCLR across the experiment

Self-identification with the avatar and threat response may have increased over the time course of the experiment (i.e., due to longer exposure or increased familiarity) and this may be related to the attenuation of the GCLR in the Body condition. To assess this possibility, we analyzed whether questionnaire ratings changed over time (across six scanning sessions). Yet there was neither a significant effect of time (Q1: $p = 0.57$, Q2: $p = 0.21$) nor a significant interaction between the effects of time and experimental condition (Body vs. No-body) (Q1: $p = 0.56$, Q2: $p = 0.19$). There also was no effect of time on GCLR ($p = 0.72$) and no interaction between time and condition ($p= 0.77$). These additional data show that the reduction of GCLR in the Body condition is independent of the time course of the experiment.

Supplementary Note 13: Other parameters that might influence GCLR

In addition, although we report a larger distance from the border in the Body condition, further analysis revealed that the central navigational preference (proposed by Kunz and colleagues, 2015) did not differ in the two conditions of the present experiment ($p = 0.84$). The undershot (drift in self-location) we observed only happened at the last moment of each navigation and was quite subtle (~1 vm on average) compared to the much larger diameter of the arena (110 vm). We also assessed correlation between GCLR and the central navigational preference but did not find a significant relationship ($p = 0.95$). The decreased GCLR can also not be explained by the difference in target locations as all objects and target locations were randomly shuffled across conditions. Additional analysis also showed that head motion artifacts did not differ between conditions (Body: 0.240±0.012 mm, No-body: 0.247±0.014 mm; $p = 0.08$) and were taken into account by the nuisance regressors with the motion parameters.

As participants were only trained in the No-body condition, it could be argued that previous exposure to the No-body, but not the Body condition, may account for the observed GCLR differences between conditions (i.e. re-exposure effect). This, however, does not seem very likely, because when reanalyzing our GCLR results across conditions, but by excluding the 1st block for each condition, we confirmed our original results and found that only the GCLR in the No-body condition was significant ($p = 6.23e-3$), whereas this was not the case in the Body condition ($p = 0.28$).

Supplementary Note 14: Hippocampal BOLD activity and compensatory mechanisms

Kunz et al. (2015) described that activation in the hippocampus might serve as a compensatory mechanism in cases where GCLR was found to be attenuated. It could therefore be argued that the present changes in GCLR across conditions may reflect similar compensatory hippocampal activity. However, hippocampal BOLD activity did not differ in the Body versus No-body condition in the present study ($p = 0.94$).

Supplementary FIGURES

Supplementary Figure 1

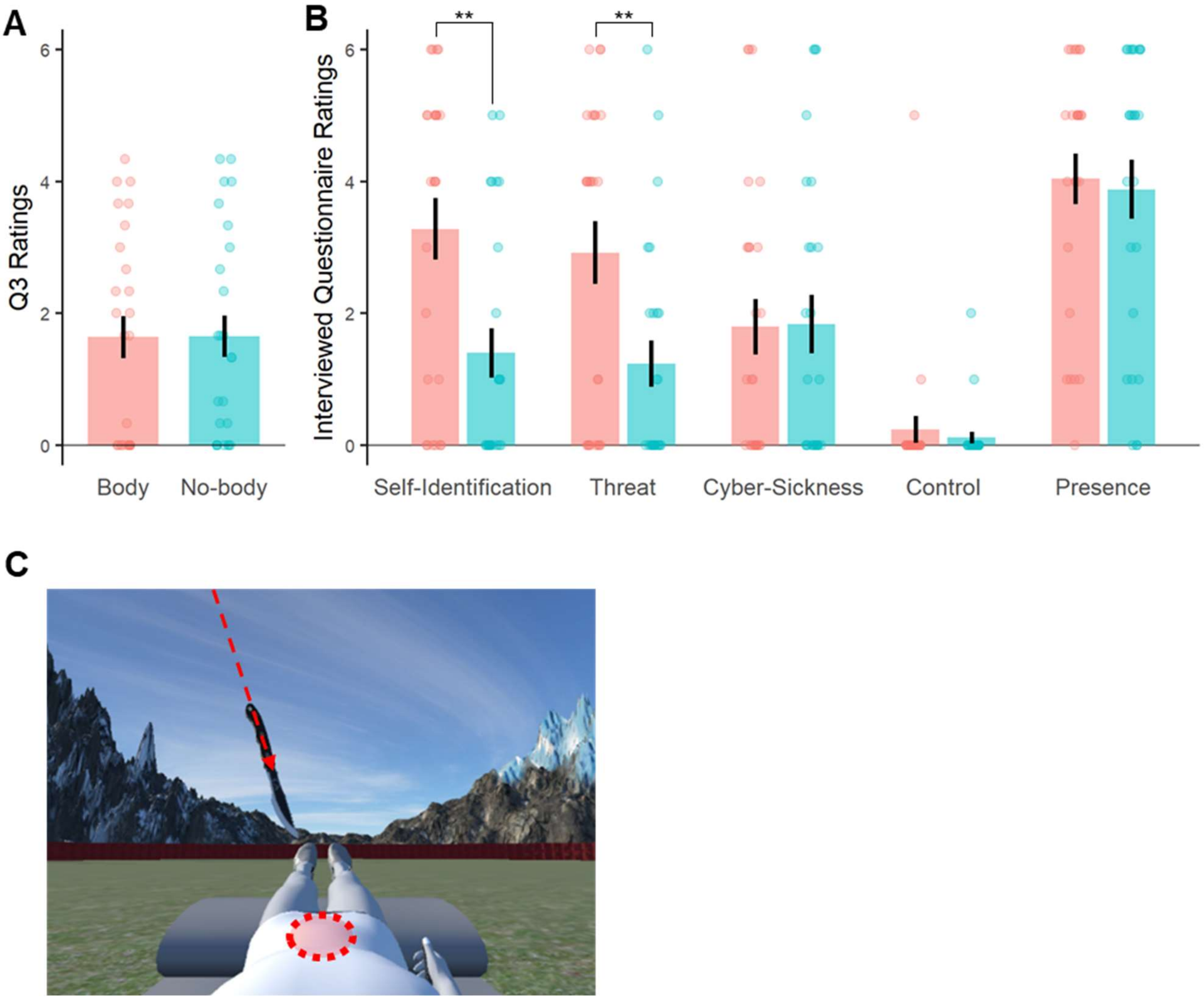

Fig. S1, related to Fig. 2 and the questionnaire section of the method. Question3 (Q3) ratings and questionnaire ratings from the post-experiment interviews. (A) Q3 ratings capturing cyber-sickness of participants did not differ between the experimental conditions ($p = 0.57$) **(B)** Verbally answered questionnaire ratings from the post-experiment interviews. The results showed the difference in 'self-identification' and 'Threat' ratings between the experimental conditions, which is in accordance with the questionnaire results answered during the task. A question for 'Presence (i.e. spatial immersion into the virtual space') was included in the post-experiment interview and did not statistically differ between the two experimental conditions. **(C)** The virtual threat (i.e. knife) was directed to the position as indicated by the red oval (red oval was not shown) in both conditions. Each error bar indicates a standard error. ** : $p < 0.01$

Supplementary Figure 2

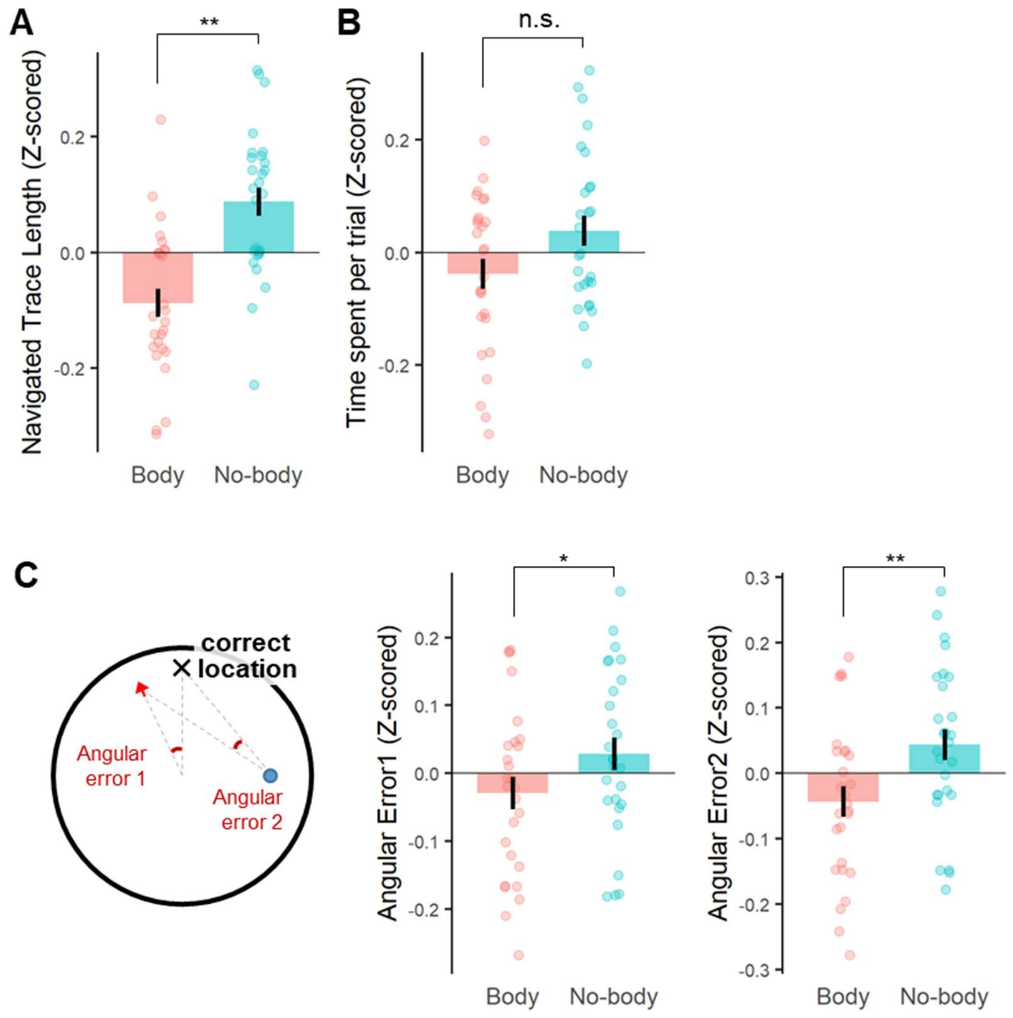

Fig. S2, related to Fig. 2,3. Spatial navigation efficiency and spatial memory precision independent of the self-drift effect were better in the Body condition (A) The participants navigated through significantly shorter traces with the embodied avatar **(B)** while they spent similar time during the trial. **(C)** Two types of angular errors calculated regardless of the distance from the border were compared between the two experimental conditions. The first type of angular error ('Angular error 1') was calculated with reference to the center of the arena (i.e., angular difference in the polar coordinate), while the second type ('Angular error 2') was computed with respect to the starting location of each trial. The results consistently showed that spatial memory precision was better (indicated by the lower angular errors) in the Body condition. Each error bar indicates a standard error. n.s. : $p >= 0.05$, * : $0.01 <= p < 0.05$, ** : $p < 0.01$

Supplementary Figure 3

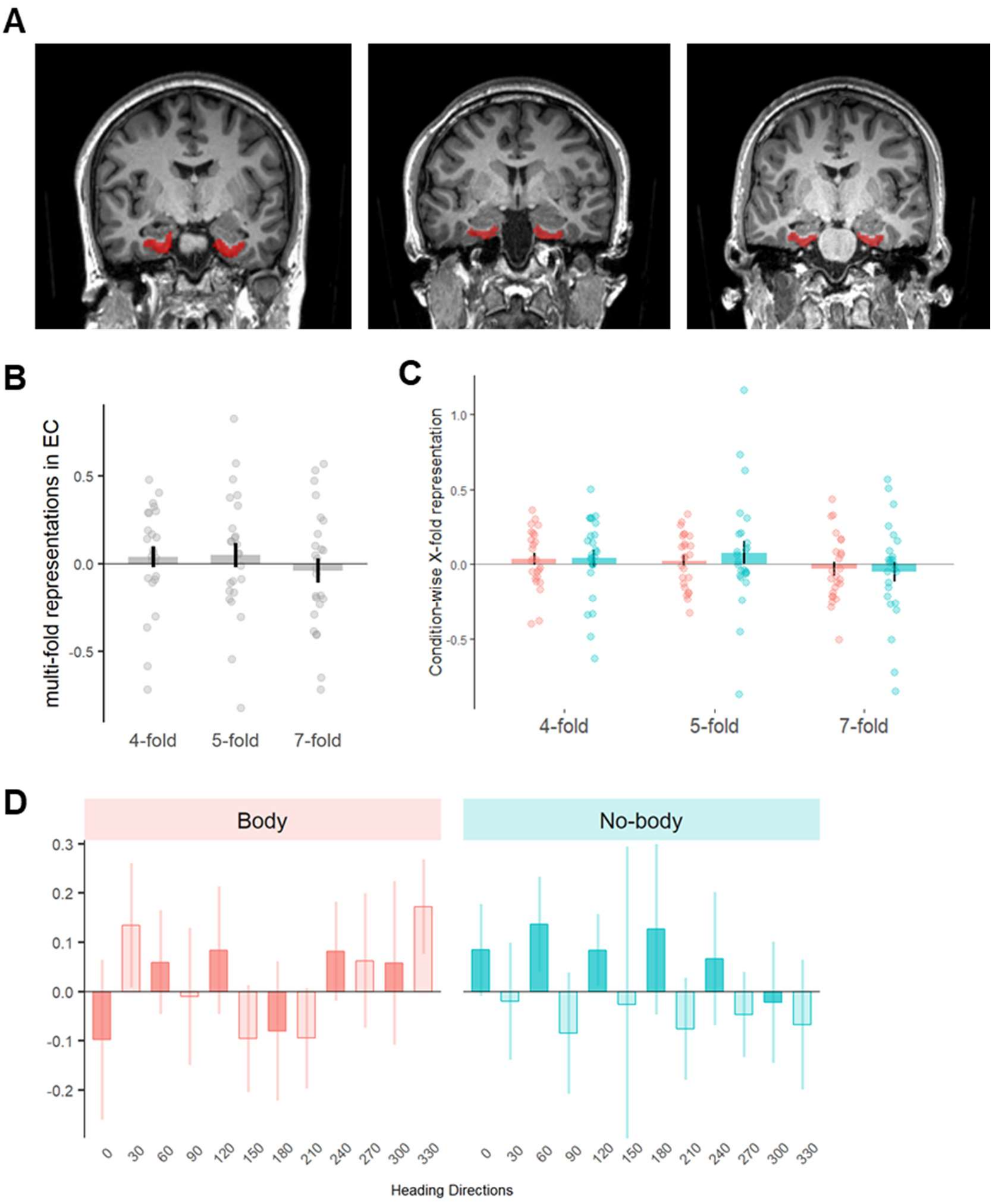

Fig. S3, related to Fig. 3. (A) EC ROIs from 3 exemplary participants, **(B)** Control multi-fold representations were not significantly greater than zero. **(C)** Condition-wise control data for multi-fold representation further confirmed that the condition-wise difference specifically occurred for the hexadirectional modulation (i.e.,6-fold). **(D)** Normalized EC activity profiles for every 30˚ heading direction showed that the typical hexadirectional modulation was hardly observable in the Body condition, while it was prominent in the No-body condition. The x-axis is relative to the estimated grid orientation. Each error bar indicates a standard error.

Supplementary Figure 4

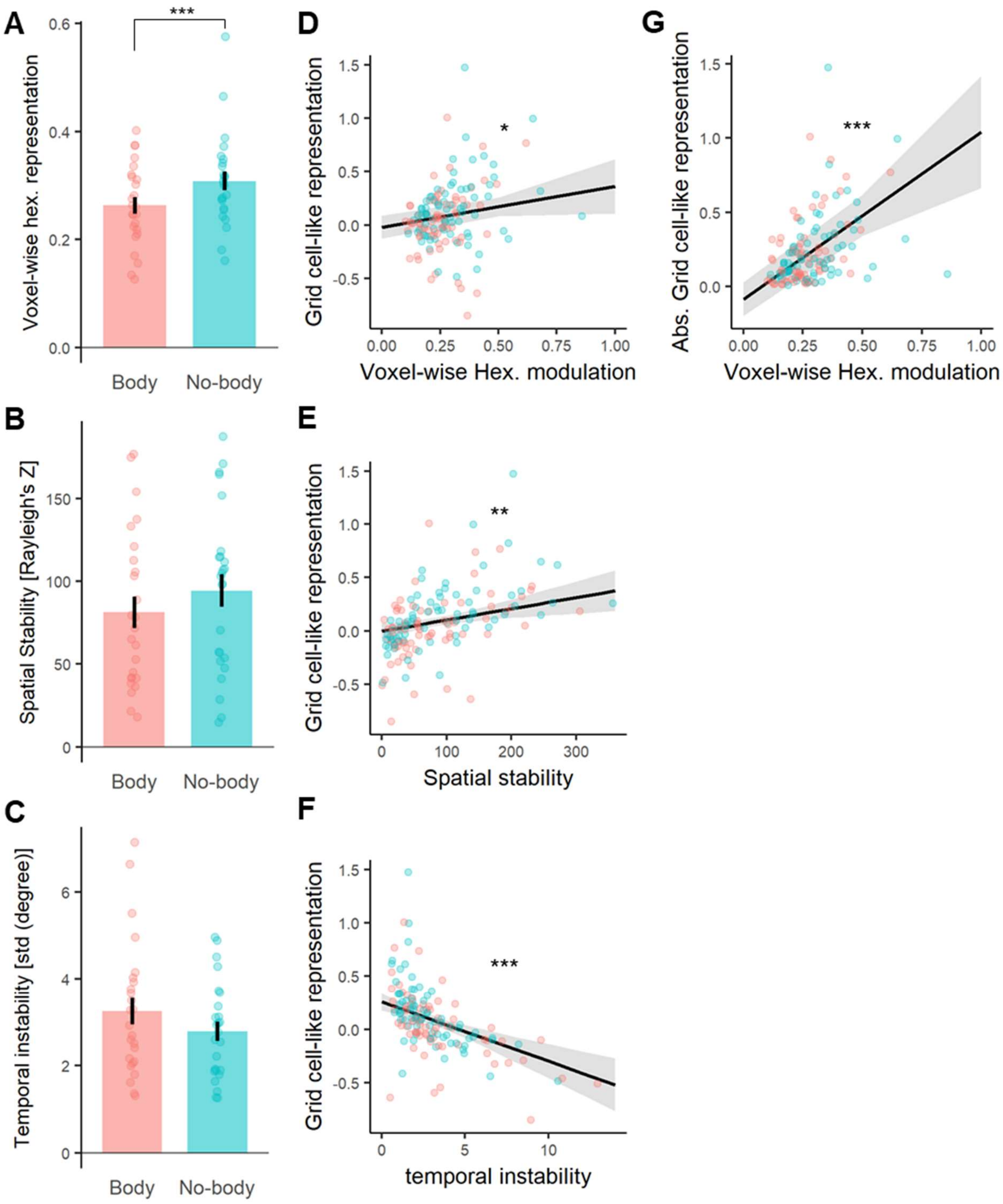

Fig. S4, related to Fig. 3. Parameters relevant to estimating Grid cell-like representation (GCLR) were consistently better in the Body condition. (A) Voxel-wise hexadirectional modulation in the entorhinal

cortex(EC) was significantly attenuated in the Body condition. **(B)** Spatial Stability (i.e. homogeneity of voxel-wise grid orientations in EC) was also lower in the Body condition, but the difference did not reach significance. **(C)** Grid orientations were less stable in time, during the Body condition. However, the difference was insignificant. **(D,E,F)** Both the voxel-wise amplitude of the hexadirectional modulation and the spatial stability were significantly and positively correlated with the estimated GCLR, while temporal instability was negatively correlated. Contributions of the three parameters(voxel-wise amplitude, spatial stability, and temporal instability) to the estimated GCLR was assessed simultaneously with a multiple mixed-effect model. **(G)** As theoretically expected, the voxel-wise amplitude of the six-fold modulation was more strongly correlated with the absolute values of GCLRs, which were less affected by wrongly calculated grid orientations. Of note, figure A-C are plotted with subject-wise mean values, while figure D-G are plotted with session-wise mean values; no data points were excluded. This was based on the statistical analysis that we applied on the data depicted respectively in each figure: 1) Wilcoxon signed-rank tests to assess the condition-wise difference and 2) a mixed effect model to assess the correlation between metrics with every round-wise value. Each error bar indicates a standard error. * : $0.01 <= p < 0.05$, ** : $0.001 <= p < 0.01$, *** : $p < 0.001$.

Supplementary Figure 5

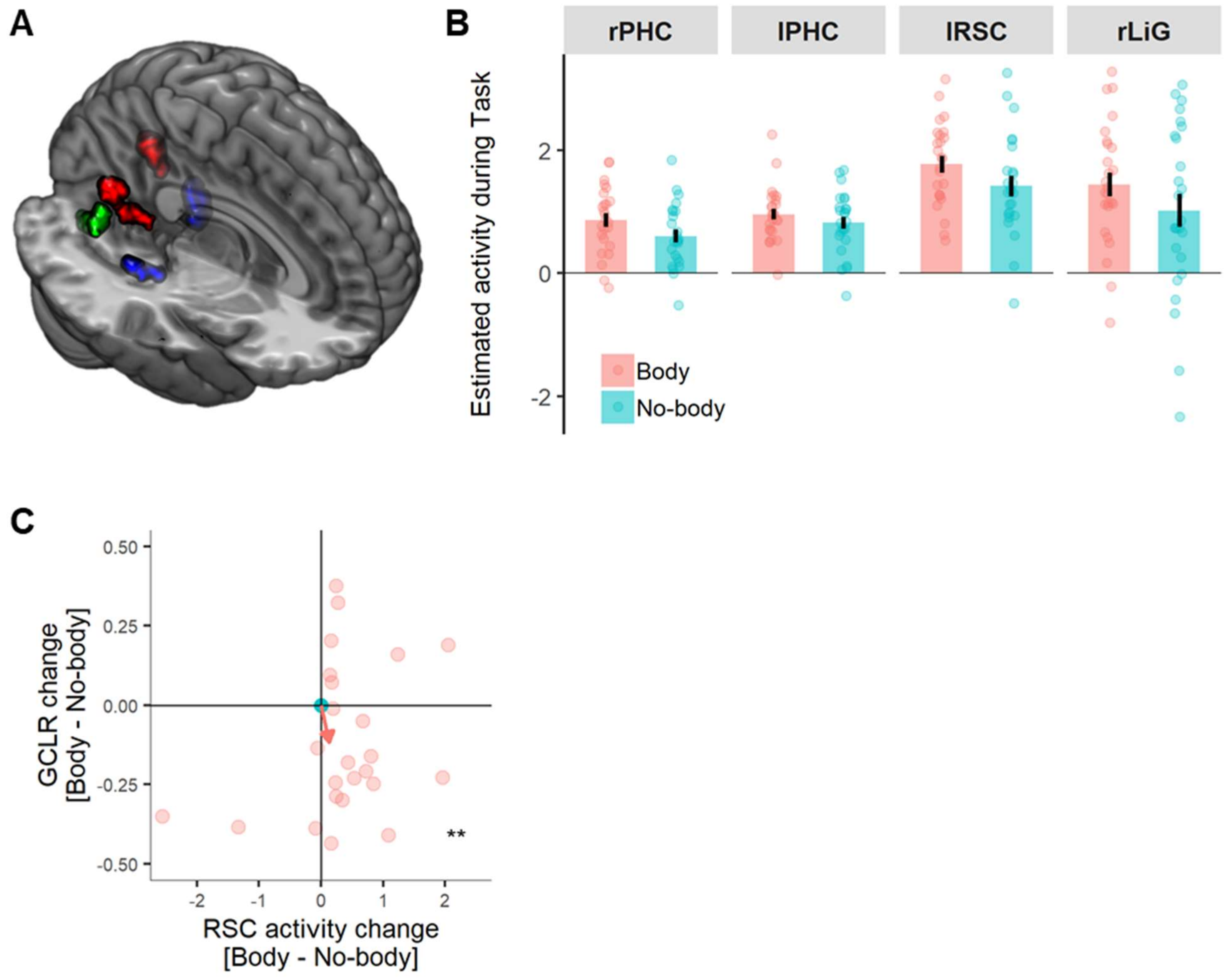

Fig. S5, related to Fig. 4. Task-related brain regions defined by the functional localizer.

(A) The bilateral parahippocampal gyrus (PHC: Blue), bilateral retrosplenial cortex (RSC: Red; see Fig. 6 for the right RSC), and right lingual gyrus (rLiG: Green) were activated during the spatial navigation task procedure. **(B)** In the four other brain regions revealed by the analysis (except for the right RSC), we could not find a significant difference between the conditions (rPHC: $r = 0.36$, $p = 0.38$ / lPHC: $r = 0.23$, $p = 1$ / lRSC: $r = 0.41$, $p = 0.21$ / rLiG: $r = 0.28$, $p = 0.83$). **(C)** Participant-wise right RSC activity changes and GCLR changes in the Body condition with respect to the No-body condition. The plot demonstrates that performing the task with a self-identified avatar reduced GCLR while strengthening the right RSC activity (multinomial test: $p < 0.001$, post-hoc binomial test: $p = 3.1e\text{-}03$, $n = 24$). The red arrow indicates mean changes across participants. Each error bar indicates a standard error. ** : $p < 0.01$.

Supplementary Figure 6

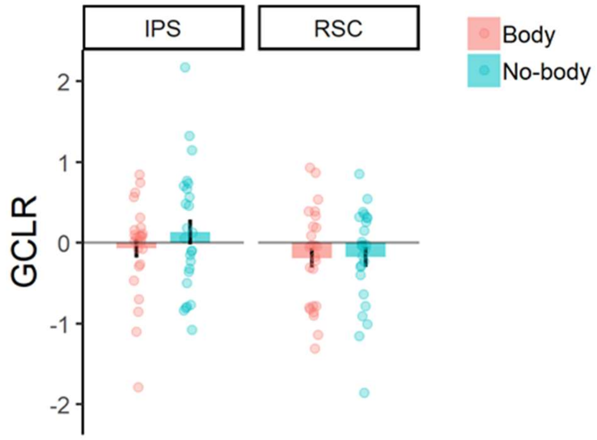

Fig. S6, related to Fig. 4. GCLRs in IPS and RSC

No significant GCLR (greater than 0) nor a significant difference between conditions was detected in either IPS or RSC. Each error bar indicates a standard error.

Supplementary Figure 7

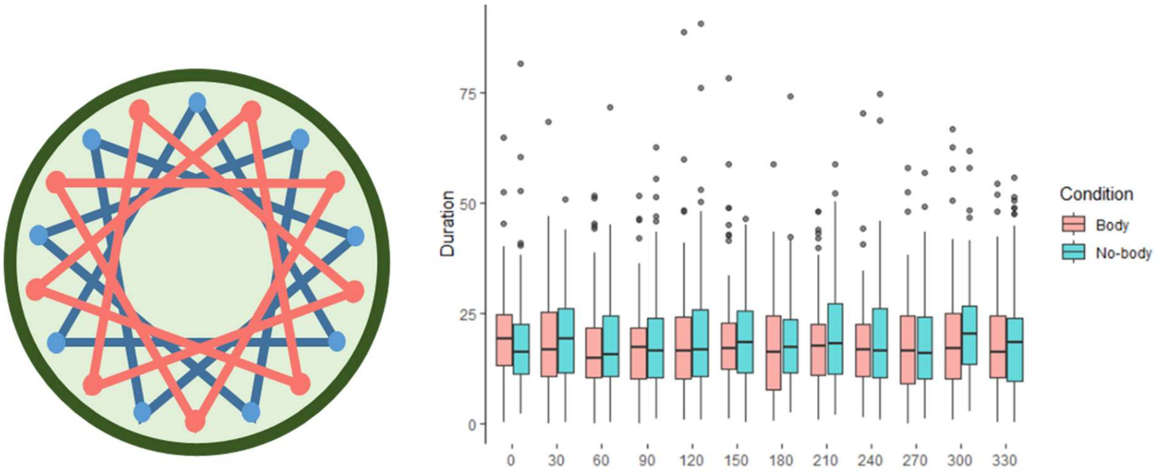

Fig. S7, related to Fig. 1 and 3. Task objects were placed to span every 20 degrees per condition during the entire experiment. The object location sets per condition were randomized across participants. Overall, our participants have navigated in various directions, which allowed the estimation of GCLR regardless of conditions. Each boxplot represents median and intraquartile range (IQR).

Supplementary Figure 8

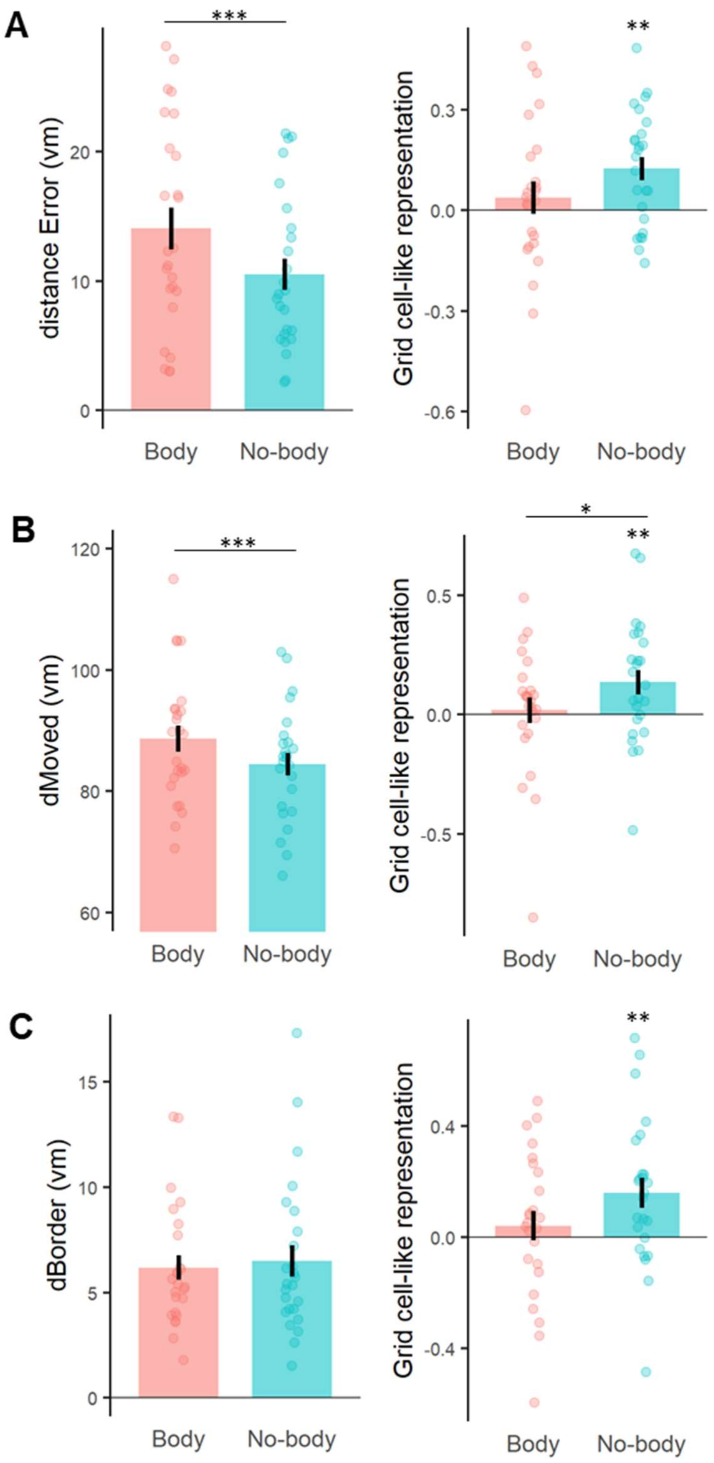

Fig. S8. Behavioral Parameters and GCLR after the resampling of the dataset to invert behavioral differences. (A) After resampling, distance error is higher in the Body condition than the No-body ($p = 2.5$ e-4). However, GCLR was still non-significant in the Body condition ($p=0.229$), while significant in the No-body condition ($p = 1.39$ e-3). This difference in GCLR between conditions did not reach significance ($p = 0.182$) **(B)** Navigated trace length (dMoved) also was inverted between conditions and was higher in the Body condition ($p = 1.03$e-4). GCLR was non-significant in the Body condition ($p = 0.121$), but significant in the No-body ($p = 7.36$ e-3). GCLRs were significantly different between conditions ($p = 0.039$). **(C)** Difference in the distance from the border (dBorder) became non-significantly different between two conditions after the resampling. GCLR was significant only in the No-body condition (Body: $p = 0.176$ / No-body: $p = 2.09$ e-3), while their difference did not reach significance ($p = 0.101$). Each error bar indicates a standard error. * : $0.01 <= p < 0.05$, ** : $0.001 <= p < 0.01$, *** : $p < 0.001$.

Supplementary Figure 9

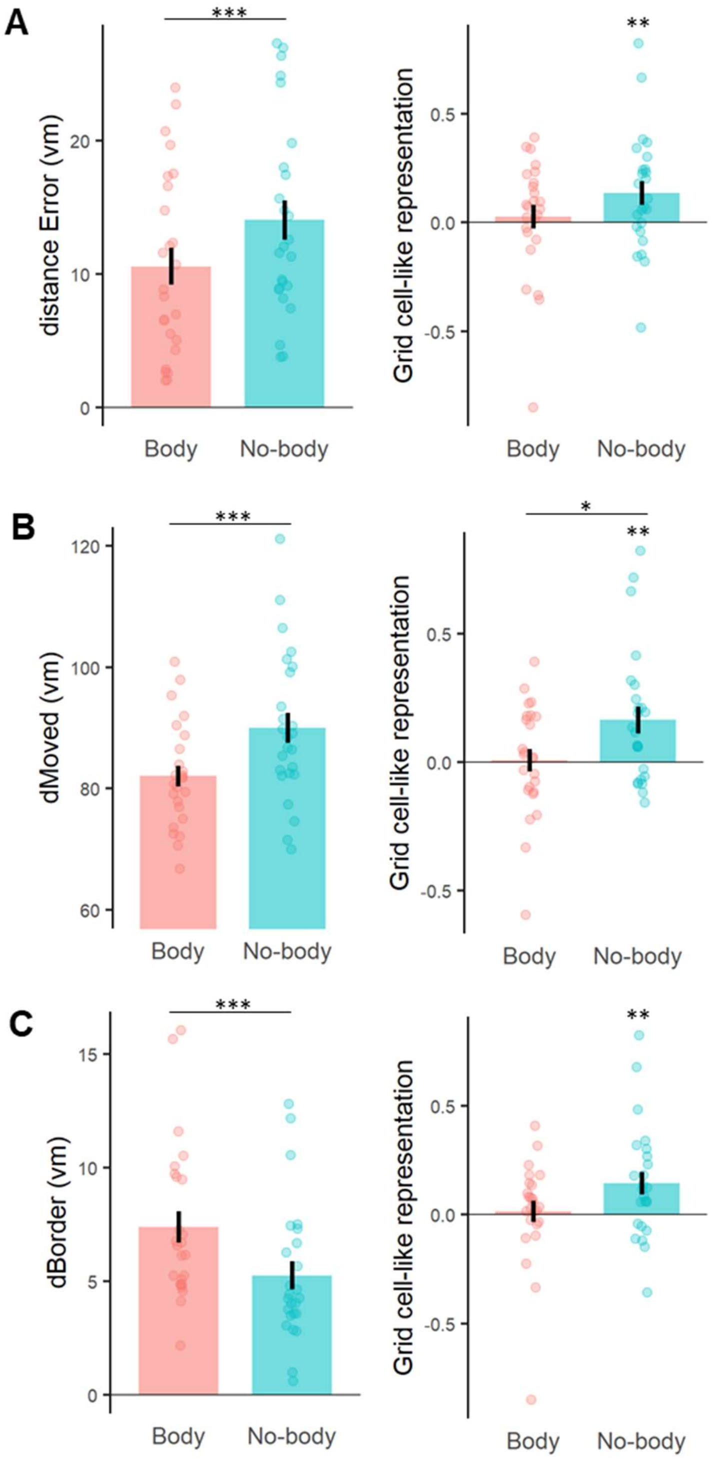

Fig. S9. Behavioral Parameters and GCLR after the resampling of the dataset to exaggerate behavioral differences. (A,B,C – left panels) Resampling analyses of the opposite direction were performed to exaggerate the existing behavioral differences (p-values for three parameters are all below 0.001; p = 1.23e-5, p = 1.19e-7, p = 8.35e-7 respectively) **(A,B,C – right panels)** Despite the opposite behavioral parameters, GCLR results from the two different resampling analyses were very similar (not different statistically). GCLRs in the Body conditions were not significant (p = 0.105 / p = 0.326 / p = 0.100, respectively). In contrast, GCLRs in the No-body conditions were significant (p = 6.78 e-3 / p = 3.07 e-3 / p = 4.03 e-3, respectively). Differences in GCLR between the condition were only significant for the dMoved resampling (p = 0.230 / p =0.034 / p = 0.107, respectively). Each error bar indicates a standard error. * : 0.01 <= p < 0.05, ** : 0.001 <=p < 0.01, *** : p < 0.001.

Supplementary Tables

Regressor	Parametric modulator	Duration per trial
Cue		2.5 s
Retrieval		variable
Retrieval	Distance error	variable
Self-Estimation		variable
Feedback		2.0 s
Collection		variable
Threat		2.5 s

Supplementary Table 1. List of regressors for whole-brain GLM analysis

Region	MNI coordinates (mm)	Cluster Size (voxels)	Peak t-value	Peak p-value
**Retrosplenial cortex / Precuneus**				
Right	20, -56, 22	121	10.67	<0.001
Left	-16, -60, 22	114	11.20	<0.001
**Parahippocampal gyrus**				
Right	24, -40, -10	58	13.21	<0.001
Left	-22, -42, -8	125	12.34	<0.001
**Lingual gyrus**				
Right	8, -70, -2	90	9.49	<0.001

$p < 0.05$, whole-brain, voxel-wise FWE correction

Supplementary Table 2. Task-related brain regions established by the functional localizer

Supplementary References

1 Doeller, C. F., Barry, C. & Burgess, N. Evidence for grid cells in a human memory network. *Nature* **463**, 657-661, doi:10.1038/nature08704 (2010).

2 Stangl, M., Shine, J. & Wolbers, T. The GridCAT: A Toolbox for Automated Analysis of Human Grid Cell Codes in fMRI. *Front Neuroinform* **11**, 47, doi:10.3389/fninf.2017.00047 (2017).

3 Kunz, L. *et al.* Reduced grid-cell-like representations in adults at genetic risk for Alzheimer's disease. *Science* **350**, 430-433, doi:10.1126/science.aac8128 (2015).

4 Stangl, M. *et al.* Compromised Grid-Cell-like Representations in Old Age as a Key Mechanism to Explain Age-Related Navigational Deficits. *Curr Biol* **28**, 1108-1115 e1106, doi:10.1016/j.cub.2018.02.038 (2018).

5 Sargolini, F. *et al.* Conjunctive representation of position, direction, and velocity in entorhinal cortex. *Science* **312**, 758-762, doi:10.1126/science.1125572 (2006).

6 Kropff, E., Carmichael, J. E., Moser, M. B. & Moser, E. I. Speed cells in the medial entorhinal cortex. *Nature* **523**, 419-424, doi:10.1038/nature14622 (2015).

7 Huffman, D. J. & Ekstrom, A. D. An Important Step toward Understanding the Role of Body-based Cues on Human Spatial Memory for Large-Scale Environments. *J Cogn Neurosci* **33**, 167-179, doi:10.1162/jocn_a_01653 (2021).

8 Huffman, D. J. & Ekstrom, A. D. A Modality-Independent Network Underlies the Retrieval of Large-Scale Spatial Environments in the Human Brain. *Neuron* **104**, 611-622 e617, doi:10.1016/j.neuron.2019.08.012 (2019).

9 Steel, A., Robertson, C. E. & Taube, J. S. Current Promises and Limitations of Combined Virtual Reality and Functional Magnetic Resonance Imaging Research in Humans: A Commentary on Huffman and Ekstrom (2019). *J Cogn Neurosci* **33**, 159-166, doi:10.1162/jocn_a_01635 (2021).

10 Maidenbaum, S., Miller, J., Stein, J. M. & Jacobs, J. Grid-like hexadirectional modulation of human entorhinal theta oscillations. *Proc Natl Acad Sci U S A* **115**, 10798-10803, doi:10.1073/pnas.1805007115 (2018).

11 Danjo, T., Toyoizumi, T. & Fujisawa, S. Spatial representations of self and other in the hippocampus. *Science* **359**, 213-218, doi:10.1126/science.aao3898 (2018).

12 Omer, D. B., Maimon, S. R., Las, L. & Ulanovsky, N. Social place-cells in the bat hippocampus. *Science* **359**, 218-224, doi:10.1126/science.aao3474 (2018).

13 Constantinescu, A. O., O'Reilly, J. X. & Behrens, T. E. Organizing conceptual knowledge in humans with a gridlike code. *Science* **352**, 1464-1468, doi:10.1126/science.aaf0941 (2016).

14 Derdikman, D. *et al.* Fragmentation of grid cell maps in a multicompartment environment. *Nat Neurosci* **12**, 1325-1332, doi:10.1038/nn.2396 (2009).

15 He, Q. & Brown, T. I. Environmental Barriers Disrupt Grid-like Representations in Humans during Navigation. *Curr Biol* **29**, 2718-2722 e2713, doi:10.1016/j.cub.2019.06.072 (2019).
