# Supplementary material for: Sense of self impacts spatial navigation and hexadirectional coding in human entorhinal cortex
Source: Commun Biol. 2022 May 2;5:406. doi: 10.1038/s42003-022-03361-5 (PMC9061856; doi:10.1038/s42003-022-03361-5)
Supplement: Supplementary file 2 — Description of Supplementary Information [file 42003_2022_3361_MOESM2_ESM.pdf]

Description of Additional Supplementary Files

File name: Supplementary Movie 1
Description: The video shows a participant's physical body (focusing on the right hand) in the scanner and the corresponding task scene while they were performing the virtual navigation task in the Body condition.
