# Supplementary material for: Sense of self impacts spatial navigation and hexadirectional coding in human entorhinal cortex
Source: Commun Biol. 2022 May 2;5:406. doi: 10.1038/s42003-022-03361-5 (PMC9061856; doi:10.1038/s42003-022-03361-5)
Supplement: Supplementary file 4 — Reporting Summary [file 42003_2022_3361_MOESM4_ESM.pdf]

nature portfolio

Corresponding author(s): Olaf Blanke

Last updated by author(s): Apr 7, 2022

Reporting Summary

Nature Portfolio wishes to improve the reproducibility of the work that we publish. This form provides structure for consistency and transparency in reporting. For further information on Nature Portfolio policies, see our Editorial Policies and the Editorial Policy Checklist.

Statistics

For all statistical analyses, confirm that the following items are present in the figure legend, table legend, main text, or Methods section.

n/a	Confirmed	
☐	☒	The exact sample size (*n*) for each experimental group/condition, given as a discrete number and unit of measurement
☐	☒	A statement on whether measurements were taken from distinct samples or whether the same sample was measured repeatedly
☐	☒	The statistical test(s) used AND whether they are one- or two-sided<br>*Only common tests should be described solely by name; describe more complex techniques in the Methods section.*
☒	☐	A description of all covariates tested
☐	☒	A description of any assumptions or corrections, such as tests of normality and adjustment for multiple comparisons
☐	☒	A full description of the statistical parameters including central tendency (e.g. means) or other basic estimates (e.g. regression coefficient) AND variation (e.g. standard deviation) or associated estimates of uncertainty (e.g. confidence intervals)
☐	☒	For null hypothesis testing, the test statistic (e.g. *F*, *t*, *r*) with confidence intervals, effect sizes, degrees of freedom and *P* value noted<br>*Give P values as exact values whenever suitable.*
☒	☐	For Bayesian analysis, information on the choice of priors and Markov chain Monte Carlo settings
☒	☐	For hierarchical and complex designs, identification of the appropriate level for tests and full reporting of outcomes
☐	☒	Estimates of effect sizes (e.g. Cohen's *d*, Pearson's *r*), indicating how they were calculated

Our web collection on statistics for biologists contains articles on many of the points above.

Software and code

Policy information about availability of computer code

Data collection	Spatial Navigation task was implemented using Unity 3D Engine (https://unity.com/). The task program is available from the corresponding author upon reasonable request.
Data analysis	MRI data was analyzed using SPM12 and gridCAT(https://www.nitrc.org/projects/gridcat) running on the Matlab R2018b.<br>Statistical assessments for behavioral and fMRI data were performed with R (v3.5.3 for Windows) and R Studio (v1.2.1335). The customized codes used for the current article are available on the open science framework (doi:10.17605/OSF.IO/U8VHA) and are open to the public.

For manuscripts utilizing custom algorithms or software that are central to the research but not yet described in published literature, software must be made available to editors and reviewers. We strongly encourage code deposition in a community repository (e.g. GitHub). See the Nature Portfolio guidelines for submitting code & software for further information.

Data

Policy information about availability of data

All manuscripts must include a data availability statement. This statement should provide the following information, where applicable:

- Accession codes, unique identifiers, or web links for publicly available datasets
- A description of any restrictions on data availability
- For clinical datasets or third party data, please ensure that the statement adheres to our policy

The data underlying the results and the main figures of this study have been uploaded on the open science framework (doi:10.17605/OSF.IO/U8VHA) and are open to the public. The raw fMRI data of this study are available from the corresponding author upon reasonable request.

Field-specific reporting

Please select the one below that is the best fit for your research. If you are not sure, read the appropriate sections before making your selection.

☒ Life sciences ☐ Behavioural & social sciences ☐ Ecological, evolutionary & environmental sciences

For a reference copy of the document with all sections, see nature.com/documents/nr-reporting-summary-flat.pdf

Life sciences study design

All studies must disclose on these points even when the disclosure is negative.

Sample size	The sampling procedure was random. No a priori statistical test was run to determine sample size. The sample sizes (n = 27) are based on the G*Power analysis reported in Nau et al., Nat Neurosci(2018) to detect significant grid cell-like representation.
Data exclusions	Two participants who entered random answers to the questionnaire excluded from the questionnaire analysis (i.e. both pressed response button repeatedly at incorrect moments during the experiment; further confirmed by post-experiment debriefing). A participant whose structural image was examined as abnormal by a medical investigator was excluded from the fMRI analyses. Another participant was excluded from the grid cell-like representation analyses due to severe image distortions and signal drop in entorhinal cortex (~2 % of voxels in the region-of-interest were above the global average). One session from a participant with head drift greater than 3 mm, but not the other sessions, was also excluded from the fMRI analyses.
Replication	No replication was attempted.
Randomization	Participants were not allocated into experimental groups.
Blinding	Participants were not allocated into experimental groups. We were aware of the experimental condition during the data collection as it is explicitly indicating by the task scene. But the task was fully automated and solely performed by the participants, avoiding any influences from the researcher.

Reporting for specific materials, systems and methods

We require information from authors about some types of materials, experimental systems and methods used in many studies. Here, indicate whether each material, system or method listed is relevant to your study. If you are not sure if a list item applies to your research, read the appropriate section before selecting a response.

Materials & experimental systems

n/a	Involved in the study	
☒	☐	Antibodies
☒	☐	Eukaryotic cell lines
☒	☐	Palaeontology and archaeology
☒	☐	Animals and other organisms
☐	☒	Human research participants
☒	☐	Clinical data
☒	☐	Dual use research of concern

Methods

n/a	Involved in the study	
☒	☐	ChIP-seq
☒	☐	Flow cytometry
☐	☒	MRI-based neuroimaging

Human research participants

Policy information about studies involving human research participants

Population characteristics	27 participants (14 females; 27 right-handed; mean age: 25.3±1.96; villagers in regional area).<br>Participants had normal or corrected-to-normal vision, and they reported no history of neurological or psychiatric disorders.
Recruitment	Participants were recruited through an online recruiting system (https://lnco-geneva.sona-systems.com/)
Ethics oversight	All procedures were done in accordance of the declaration of Helsinki (2003) and approved by the local ethics committee (Commission Cantonale d'Ethique de Genève), IRB #: GE 15-273

Note that full information on the approval of the study protocol must also be provided in the manuscript.

Magnetic resonance imaging

Experimental design

Design type: Task with event-related and block design. (Events happened in blocks with different conditions)

Design specifications: There were 6 blocks composed of 14 trials each per participants. 3 blocks were assigned to each condition by the pseudo-randomized order (A-B-B-A-A-B). The task was self-paced, the duration of each block varied depending on the participant's performance (mean: 9.0 ± 0.70 min), but didn't differ between the conditions.

Behavioral performance measures: Distance error (distance between retrieved and correct locations), total navigated distance, and task time during the navigation task was recorded for each trial. Mixed-effects regressions were respectively used to assess their differences between conditions. Trials with too short task time (< 1s) or navigated distance (< 5vm) were regarded as a manipulation error and excluded from the analyses.

Acquisition

Imaging type(s): Functional

Field strength: 3T

Sequence & imaging parameters: T2*-weighted Echo Planar Imaging (EPI) sequence with the following parameters: TR = 1000 ms, TE = 32 ms, Slice thickness = 2mm (no gap), In-plane resolution = 2 mm x 2 mm, Number of slices = 66, Multiband factor = 6, FoV = 225 mm, Flip angle = 50°, slice acquisition order = interleaved.

Area of acquisition: Whole-brain

Diffusion MRI: ☐ Used ☒ Not used

Preprocessing

Preprocessing software: Using SPM 12, functional images were slice-time corrected, realigned and unwarped and co-registered with the anatomical image. All preprocessed functional images were smoothed with a 5mm full-width-half-maximum Gaussian smoothing kernel. Freesurfer v6.0.0 was used for the automated brain parcellation on the anatomical images.

Normalization: For the whole brain GLM analysis, normalization to MNI space was performed by a 12 parameter affine transformation and non-linear transformation with SPM12.

Normalization template: ICBM space template - European brains

Noise and artifact removal: Motion parameters were calculated during realignment in SPM and visualized with ArtRepair Toolbox(v4) to manually detect severe motion artifacts. The detected motion artifacts were taken into account by additional GLM regressors.

Volume censoring: N/A

Statistical modeling & inference

Model type and settings: Univariate model (multiple regression).
For the analysis of grid cell-like representation, stand and navigation condition with two parametric regressors consist of the first GLM model to calculate putative grid-orientation. Based on it, in the following GLM, grid cell-like representation was respectively assessed (1) by the six-fold rotation symmetric sinusoidal parametric regressor and (2) by the contrast of beta estimate between aligned vs. misaligned navigation. (see Method for the details)
For the analysis of task-related activity, task phases (Cue, Retrieval, Self-Estimation, Feedback..) composed first level GLM model. In the second level GLM, brain regions activated during the task phases were estimated by the contrast between corresponding beta estimate and implicit baseline. (See the Methods and supplementary table 1 for the details)
For GLM, six motion parameters from the realignment step and additional regressor(s) indicating significant motion artifact were included as multiple regressors.

Effect(s) tested: To confirm grid cell-like representation in the entorhinal cortex, the corresponding beta estimates were assessed whether greater than '0' with one-sided Wilcoxon signed rank test. For the comparison between the conditions, beta estimates were compared with paired two-sided Wilcoxon signed rank test (see Methods).

Specify type of analysis: ☐ Whole brain ☐ ROI-based ☒ Both

Anatomical location(s): Entorhinal Cortex (generated by Freesurfer based on the Desikan-Killiany Atlas)
Intraparietal sulcus (generated by SPM anatomy toolbox in the MNI space)

Statistic type for inference (See Eklund et al. 2016): Voxel-wise tests

Correction: FWE

Models & analysis

n/a	Involved in the study
☒	☐ Functional and/or effective connectivity
☒	☐ Graph analysis
☒	☐ Multivariate modeling or predictive analysis
